# Metabolic adaptation to vitamin auxotrophy by leaf-associated bacteria

**DOI:** 10.1038/s41396-022-01303-x

**Published:** 2022-08-20

**Authors:** Birgitta Ryback, Miriam Bortfeld-Miller, Julia A. Vorholt

**Affiliations:** Institute of Microbiology, ETH Zurich, 8093 Zurich, Switzerland

**Keywords:** Microbial ecology, Microbiology

## Abstract

Auxotrophs are unable to synthesize all the metabolites essential for their metabolism and rely on others to provide them. They have been intensively studied in laboratory-generated and -evolved mutants, but emergent adaptation mechanisms to auxotrophy have not been systematically addressed. Here, we investigated auxotrophies in bacteria isolated from *Arabidopsis thaliana* leaves and found that up to half of the strains have auxotrophic requirements for biotin, niacin, pantothenate and/or thiamine. We then explored the genetic basis of auxotrophy as well as traits that co-occurred with vitamin auxotrophy. We found that auxotrophic strains generally stored coenzymes with the capacity to grow exponentially for 1–3 doublings without vitamin supplementation; however, the highest observed storage was for biotin, which allowed for 9 doublings in one strain. In co-culture experiments, we demonstrated vitamin supply to auxotrophs, and found that auxotrophic strains maintained higher species richness than prototrophs upon external supplementation with vitamins. Extension of a consumer-resource model predicted that auxotrophs can utilize carbon compounds provided by other organisms, suggesting that auxotrophic strains benefit from metabolic by-products beyond vitamins.

## Introduction

Coenzymes are essential for cellular metabolism. They form the core of many enzyme-catalyzed reactions (e.g., redox reactions, transaminations, and carbon–carbon bond formation), and act as carriers for one-carbon units and organic acids [1]. Organisms with defects in their biosynthesis, auxotrophs, must obtain coenzymes or coenzyme precursor molecules, i.e., vitamins by external supply. Because they rely on the supply of vitamins in their environment to enable growth, coenzyme instability would exacerbate the need for external supply. It has recently been shown that the carbon backbone of coenzymes is stable in vivo [2]. In fact, coenzyme longevity, i.e., the absence of intracellular degradation and rebuilding, is an inherent property that might have been selected for evolutionarily [2, 3] and can be considered a prerequisite for the proliferation of auxotrophic organisms. Auxotrophic requirements can evolve rapidly in laboratory evolution experiments in nutrient-rich media as shown in *Escherichia coli* populations [4]. Auxotrophic strains have also been isolated from nature and are often the result of gene loss [5–7]. However, it is currently unclear how common auxotrophies are in bacteria in most environments. A number of recent studies have reported predictions on nutritional requirements directly from genomic data using either genome-scale metabolic models or identifying gaps in genomes without experimental validation [8–13] or focusing the experimental validation to few strains [14–16]. These studies have been used to predict interaction networks of microbial communities, which in turn have served to infer metabolic cross-feeding in bacterial communities [17]. The frequency of auxotrophies for coenzymes, nucleobases, and amino acids in sequenced bacteria has been estimated in computational analyses and could be as high as 75% [13], although these analyses likely exhibit a high false positive rate when compared to experimentally obtained data [18].

Auxotrophs require an external supply of vitamins, which raises the question of physiological or other adaptations of auxotrophs, including vitamin storage. It has long been known that auxotrophic lactic acid bacteria maintain growth after the removal of vitamins [19]. Although intracellular storage of elemental compounds such as carbon, phosphorus, and nitrogen has been shown [20–22], the storage of small molecules—including vitamins and coenzymes—is currently not well established. It is also not clear how widespread potential storage is in environmental bacteria. There is a renewed interest to assess storage of diverse compounds due to the ecological implications of microbes’ ability to maintain growth without external access to resources [23]. Prototrophic *E. coli* strains that were mutated in genes involved in biosynthesis of coenzymes, i.e., “artificial” auxotrophic mutants, have a storage capacity for only about one doubling after removal of the essential vitamin [2]. This two-fold excess pool of coenzymes is probably the minimum requirement to keep metabolism robust to cell expansion during cell division. These results led us to hypothesize that for auxotrophs found in nature where coenzyme supply may be erratic, different strategies to cope with nutrient dependence and coenzyme storage may have evolved. Loss of biosynthesis of one compound could also affect a cell’s function in a more systemic way. Direct physiological consequences resulting from loss of biosynthesis have been studied in model organisms such as *E coli*, *Bacillus subtilis*, and *Acinetobacter baylyi* and include fitness benefit [4, 13, 24] and cross-feeding potential [25, 26]. These studies show that, as long as the required nutrient is available, auxotrophs outcompete the wild-type. It can be anticipated that given sufficient evolutionary time, other traits might be selected for to compensate for the loss of a biosynthetic ability. This loss of an essential function, e.g., a particular biosynthetic pathway, may then extend to other compounds that are available in the now obligatory niche, as well as to features that help deal with nutrient shortage. The latter could involve, for example, optimizing the use of auxotrophic coenzymes in metabolic pathways, increased vitamin storage, investing more energy into repair systems, or optimizing growth and nutrient uptake rates [9, 27–29].

Here, we first investigate the occurrence of auxotrophies in the *At*-LSPHERE collection, which consists of 224 strains isolated from leaves of *Arabidopsis thaliana* plants growing in nature [30]. The strains had been isolated on vitamin and amino acid replete medium, which allows for systematic nutrient drop-out experiments to examine auxotrophies within the strain collection. We then address whether auxotrophic strains maintain a higher coenzyme storage than prototrophic strains and explore genomic traits that co-occur (and potentially co-evolved) with auxotrophy. Finally, we examine vitamin acquisition as well as auxotrophic fitness in co-culture experiments.

## Results

### Vitamin storage in auxotrophic bacteria

**Fig. 1:**
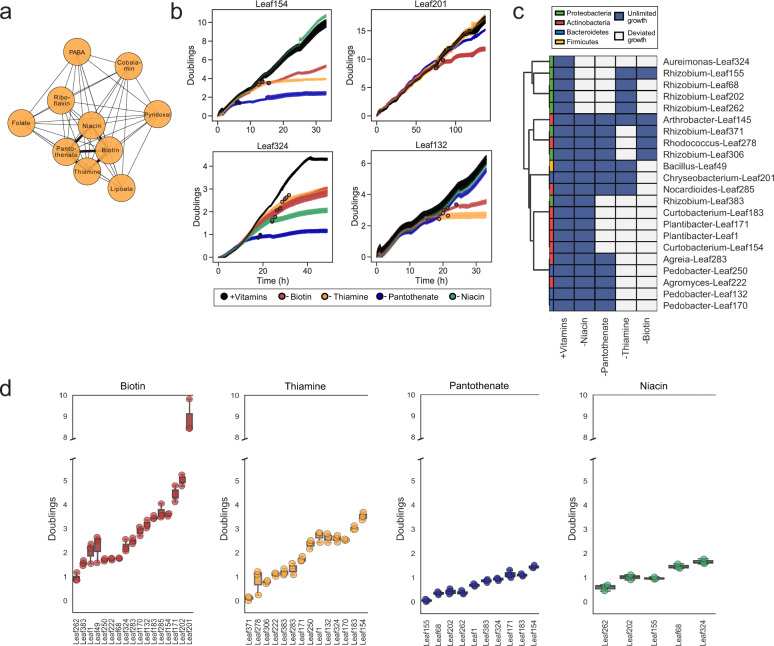
Vitamin storage in auxotrophic strains. **a** Co-occurring vitamin auxotrophies as inferred from lack of growth without a given vitamin in a screen on drop-out media for 50 strains. Niacin, pantothenate, biotin, and thiamine were the most commonly required vitamins. Edge thickness correspond to the number of strains in which the two putative auxotrophies co-occur. PABA = para-aminobenzoic acid. (Source data in Source Data 1.) **b** Representative dynamic vitamin depletion experiments. Growth is presented as doublings over time. At *t* = 0, a switch from preculture supplemented with all four vitamins to a vitamin-deplete medium (coloured lines) or, as a control, supplemented medium (black line) was performed. If a strain was auxotrophic for a given vitamin, it depleted off that vitamin after some time and stopped growing; for example, *Aureiomonas* Leaf324 was found auxotrophic for all four vitamins. The time point at which vitamin-depleted and supplemented cultures deviated are visualized over the lineplot for all three replicates separately (see Materials and Methods for details). (Source data in Source Data 2.) **c** Auxotrophy heatmap. Light color refers to compounds without which a strain's growth deviates from the supplemented control and thus the strain is auxotrophic for. For example, for *Aureiomonas* Leaf324, all 4 depletion columns are light grey as its growth ceased without each of the compounds individually. (Source data in Source Data 3.) **d** Coenzyme storage in auxotrophic strains (interquartile range and median). The number of doublings in the absence of the indicated vitamin corresponds to the storage capacity. (Source data in Source Data 3). Source Data 1Source Data 2Source Data 3Source Data 3.

To identify auxotrophic strains for this study, we screened a representative 224-member strain collection (*At*-LSPHERE) [30]. Briefly, triplicates of each strain were cultivated in 96-well plates filled with liquid media supplemented with vitamins or amino acids, with both types of compounds, or without supplements. High-throughput optical density (OD_600_) measurements were used as a readout for growth. We found that out of the 156 strains that grew in the medium, 50% (78) required supplements for growth and were therefore likely auxotrophic (Supplementary Fig. 1). Auxotrophy was especially common in Actinobacteria and Alphaproteobacteria, of which more than half of the tested strains were auxotrophic. From these 78 strains, we selected 50 putative auxotrophs for further characterization in individual drop-out media and found that most vitamin auxotrophies were for biotin, thiamine, niacin, and pantothenate for which we found 10 or more auxotrophs each (Fig. 1a; Supplementary Fig. 1).

Although it is useful to characterize nutrient requirements of many strains in parallel, the application of a screen to elucidate the nutrient requirements of individual strain is limited due to linear range of plate readers, which could lead to false predictions. To overcome this limitation and for further analysis, we selected 22 strains from various phyla that did not form aggregates upon growth in liquid media, which we ensured visually and by confirming that their colony-forming unit counts were within the expected range (order of 1e9/ml) (Supplementary Table 1; see selected strains in Supplementary Table 2). We cultivated each strain in semi-continuous batch cultures and monitored OD_600_ at 10 minute intervals, diluting each well with fresh medium after every two doublings to ensure continued exponential growth. We used these data to examine growth of each of the strains on glucose minimal media supplemented with biotin, niacin, thiamine, and pantothenate, and observed that all strains grew in the presence of these four vitamins (black trajectories in Fig. 1b and Supplementary Fig. 2). Subsequently, we performed growth experiments by switching cells to vitamin-free media (see Methods), thereby exposing the cells to vitamin depletion. We classified a given strain as an auxotroph if its growth in a vitamin drop-out medium deviated from the supplemented control cultures (scatterplot overlay in Fig. 1b and Supplementary Fig. 2). All 22 strains were thereby confirmed to be auxotrophs in this experiment (Fig. 1c). Specifically, each strain was auxotrophic for two vitamins on average, and 80% of the strains were auxotrophic for multiple vitamins.

Inside cells, vitamins are processed into coenzymes. Coenzymes are catalytically highly reactive, yet stable [2]. Because they are not consumed in metabolic reactions and are only synthesized to compensate dilution by growth, coenzymes or their precursors are potentially storable in cells. To assess the potential for intracellular vitamin storage in the selected auxotrophic strains, we used the semi-continuous growth data to calculate the total number of doublings each strain achieved after vitamin deprivation before deviating from exponential growth as determined by comparison to supplemented control cultures (Fig. 1b; Supplementary Fig. 2). These values can thus serve as indicators of vitamin storage. We found that most strains had a vitamin storage allowing for 1–2 doublings, which was similar to that of *E. coli* mutants [2]. However, we also observed that some auxotrophic strains maintained exponential growth for a prolonged period of time after the removal of biotin, translating to 4–5 doublings using the biotin reserves (16–32 fold excess pool). One strain, *Chryseobacterium* Leaf201, had a reservoir that allowed for 9 doublings (512 fold excess) after vitamin withdrawal (Fig. 1d). We also exemplarily confirmed for *Rhizobium* Leaf68 that the growth arrest coincides with intracellular depletion of the coenzyme (Supplementary Fig. 3).

### Gene absence underlies auxotrophy

**Fig. 2:**
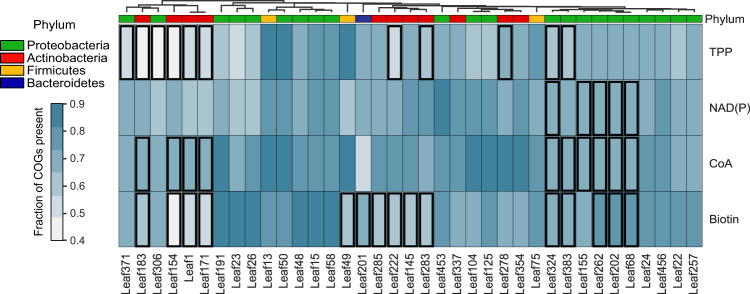
Identification of mutations underlying auxotrophy. Source data in Source Data 4. **a** Fraction of COG terms of each coenzyme biosynthesis pathway (rows) present in each strain (columns); strains are color-coded by phylum and clustered by percentage of pathway present. Black rectangles indicate that the corresponding strain was found to be auxotrophic for the coenzyme in this study (Fig. 1).

Knowing vitamin requirements for 22 validated strains, we next set out to identify the potential genetic basis for the observed auxotrophies by analysing the vitamin biosynthetic pathways using Clusters of Orthologous Groups of Proteins (COG) annotation. As prototrophic controls, we studied additional 13 strains that we confirmed to grow on minimal medium without supplements (Supplementary Fig. 4). First, we asked if we could have predicted the observed auxotrophy from genomic data alone. Following previous reports in which the percentage of biosynthetic genes absent from a pathway has been used as a marker of auxotrophy [12–15, 31], we studied the fraction of biosynthetic COG terms present on all four coenzyme synthesis pathways. All strains, including prototrophs, lacked many biosynthetic steps in the pathways for all four studied coenzymes (biotin, thiamine, coenzyme A, NAD), and we did not observe systematically fewer pathway steps in auxotrophs (Fig. 2). We then asked whether inferring auxotrophies from the presence/absence of individual genes would result in improved prediction of experimentally validated auxotrophies. To this end, we compared the frequency at which each COG term of a vitamin biosynthesis pathway was present for the auxotrophic versus prototrophic group using a Chi-squared test. In this way, we identified 1–3 absent genes sufficient to explain each auxotrophy (Table 1). For more detail on the nature of the specific reactions, see Supplementary Note 1.

**Table 1:** Genes mapping to COG terms that are significantly less prevalent in auxotrophs vs. prototrophs.

Function	COG	Present (%)		Strains		Vitamin	*q* value
		Auxotrophs	Prototrophs	Auxotrophs	Prototrophs		
Dethiobiotin synthetase BioD	COG0132	0	76	18	17	Biotin	7.13e−4
Biotin synthase BioB	COG0502	22	76	18	17	Biotin	8.65e−2
Aspartate oxidase NadA	COG0379	0	83	5	30	Niacin/NAD	5.73e−2
Quinolinate synthase NadB	COG0029	0	87	5	30	Niacin/NAD	3.68e−2
Nicotinate-nucleotide pyrophosphorylase NadC	COG0157	0	87	5	30	Niacin/NAD	3.68e−2
Pantothenate synthetase PanC	COG0414	10	84	10	25	Pantothenate/CoA	1.66e−2
Ketopantoate hydroxmethyltransferase PanB	COG0413	10	84	10	25	Pantothenate/CoA	1.66e−2
Thiazole synthase ThiG	COG2022	14	100	14	21	Thiamine/TPP	1.26e−5
Thiamine biosynthesis protein ThiC	COG0422	7	100	14	21	Thiamine/TPP	1.26e−5

Source data in Source Data 4. *p* values were computed via χ^2^ tests and corrected with FDR. The distribution of each of the COG term in the table within the complete 224 member *At*-LSPHERE community is shown in Supplementary Fig. 6.

After identifying 1–3 genes likely responsible for each observed auxotrophy, we investigated whether the findings also applied to the remaining auxotrophic strains in the *At*-LSPHERE strain collection (Supplementary Fig. 1c and Fig. 1a). To achieve this, we wanted to create robust classifiers for each of the four observed auxotrophic requirements. We first generated a sample of 63 auxotrophic strains, for 35 of which were validated above and additional 28 strains for which predictions from Supplementary Fig. 1c were used as the target variable. Secondly, we trained a decision tree classifier based on either the 1–3 features selected in the chi-squared analysis (Table 1) or equally many randomly selected features. These models reached performance metrics of 80–100% (Supplementary Fig. 5). We used the models to predict auxotrophy for 139 strains from the *At*-LSPHERE collection for which growth was observed in the supplemented medium (and therefore are comparable in physiology), and for which COG annotations were available (Supplementary Table 3). We also used these features to reinvestigate auxotrophy at the taxonomic level. The COG terms we identified to be absent in the analysis reproduced the estimated phylogenetic enrichment for auxotrophy in Actinobacteria and Alphaproteobacteria closely related to *Rhizobium* spp. (Supplementary Fig. 6; compare to Supplementary Fig. 1).

### Genome reduction in auxotrophic strains is mainly non-specific

**Fig. 3:**
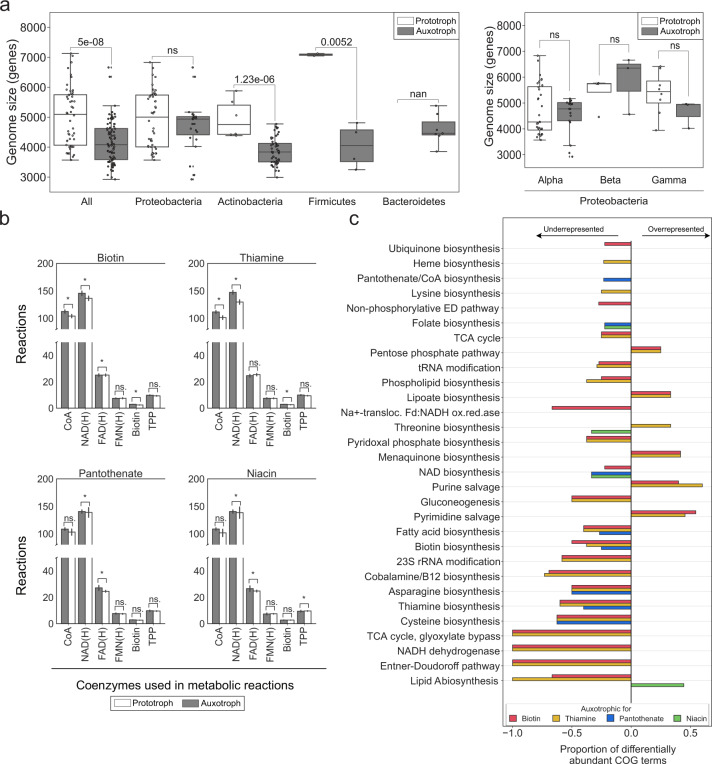
Genome-wide comparative genome analysis between prototrophs and auxotrophs. Genomes obtained from RefSeq with the accession numbers in Source Data 5. In all panels, 139 strains that grew in liquid cultures (Supplementary Fig.1) and for which COG annotations were available were included. Auxotrophy status was predicted using the models in Fig. 2 and Table 1. **a** Genome sizes between auxotrophs and prototrophs. **b** Number of reactions that require each vitamin-derived coenzyme (*x*-axis) in strains that are auxotrophic vs. prototrophic for each coenzyme (in separate plots). For example, the first plot at the top left shows a comparison of coenzyme usage of 6 coenzymes between biotin auxotrophs and prototrophs. Metabolic models were obtained by executing CarveMe [33] on the RefSeq accession numbers. **c** Pathways on which >20% of genes were differentially present between auxotrophs and prototrophs for each vitamin. Here, “underrepresented” refers to less abundant in auxotrophs. Source Data 5.

Genome reduction has previously been described in environmental auxotrophic bacteria [32]. We thus addressed genome reduction and potential concomitant gene loss events for the 139 strains for which auxotrophy predictions were possible based on functional genomic annotation and observed growth in a defined medium (Supplementary Fig. 6). Indeed, we found significantly smaller genomes in auxotrophs when compared to prototrophs (Fig. 3a). This effect was nonetheless class/phylum dependent: Genome reduction was significant in Actinobacteria (19% smaller genomes), whereby a high fraction (>50%) of strains are auxotrophs (Supplementary Fig. 1b). As there are only six species of Firmicutes within the analysed strains, drawing general conclusions is challenging. However, the four auxotrophs exhibit a genome that was only half the size of the two prototrophic strains and also feature fewer open reading frames (Supplementary Fig. 7). The auxotrophic strains identified here required on average two amino acids and two vitamins (Fig. 1, Supplementary Fig. 1). Therefore, the observed loss of biosynthetic genes would account for 0.25% smaller genomes in auxotrophs (for a bacterium that encodes on average 5,000 genes; estimated based on Fig. 3a). Thus, our observation that auxotrophs have smaller genomes provokes the question if there are other auxotrophy-associated biological processes or pathways that explain the genome reduction.

A conceivable genomic adaptation that would link auxotrophy to genome reduction is that auxotrophic strains have evolved to be less dependent on coenzymes that they cannot synthesize. However, the opposite scenario is also plausible: if the vitamin is freely available in the environment, there could be a positive selection pressure to prefer coenzymes from costless vitamins. To investigate whether there is a correlation (positive or negative) between auxotrophy for a given coenzyme and use of the same in terms of number of enzymes, we created genome-scale metabolic models for all individual strains (GEM) [33]. Overall, the number of reactions in metabolic models were similar in both groups (Supplementary Fig. 7), indicating that auxotrophs do not have streamlined metabolic networks. We set out to investigate whether auxotrophic strains preferentially use one coenzyme over the other in their enzymatic reactions; in particular, niacin auxotrophs may potentially use FAD or FMN dependent enzymes instead of NAD(P) dependent ones. Using GEMs, we computed the number of enzymes that require each coenzyme for both auxotrophs and prototrophs to measure the degree of dependency that a strain has on the corresponding vitamin. Although GEMs do not include information about expression levels of the individual enzymes in question (and therefore potential differential requirement of the enzyme), their stoichiometric constraints and network structure provide greater confidence for gene expression than genomic analysis alone. We found that for most coenzymes, auxotrophs and prototrophs had the same number of reactions (Fig. 3b). Niacin auxotrophs had 10% more enzymes using NAD(H) and 20% less enzymes using FAD(H) (*p-*value <0.05, χ^2^ test), indicating a preference to use the coenzyme for which the biosynthesis is lost. For biotin, auxotrophic strains had 15% more biotin-dependent enzymes (*p-*value <0.05, χ^2^ test). Overall, this analysis shows that the genome reduction observed in auxotrophic strains cannot systematically be explained by fewer enzymes that require the respective coenzyme. As such, metabolic capacity may not be reduced in auxotrophs, and auxotrophs may have allocated their metabolic reactions to using the essential vitamins.

Apart from direct impact on the use of coenzymes, we reasoned that auxotrophic strains might have evolved other traits indirectly related to coenzyme usage, either prior to or after becoming auxotrophs. Such traits could entail loss of function events in other biosynthesis pathways, as well as loss of genes that are not essential in the confined niche, such as utilization of a carbon source. We therefore, sought to address metabolic reallocation more generally. To find genes whose presence correlate with auxotrophy, we performed a genome-wide functional genomics analysis. We first evaluated whether each COG term was differentially abundant between auxotrophs and prototrophs, then mapped the significantly different COG terms to pathways. Significance was determined by χ^2^ test and multiple testing correction was performed with FDR. In this way, we identified a set of conserved differences between auxotrophs and prototrophs (Fig. 3c, Supplementary Table 4). Overall, the pattern was dominated by loss of function in auxotrophs. Setting the cut-off of differentially abundant COG terms to 20% of all pathway genes, we recovered the biosynthetic pathway for each known auxotrophy, which confirmed that this approach can capture biologically meaningful differences between the groups. Other gene absences entailed loss of enzymatic function in energy, especially glucose metabolism as well as other biosynthetic pathways such as amino acids and other coenzymes beyond the ones tested here, whereas salvage systems were more prevalent in the auxotrophic group.

### Auxotrophs are complemented by vitamins from co-cultivated species

**Fig. 4:**
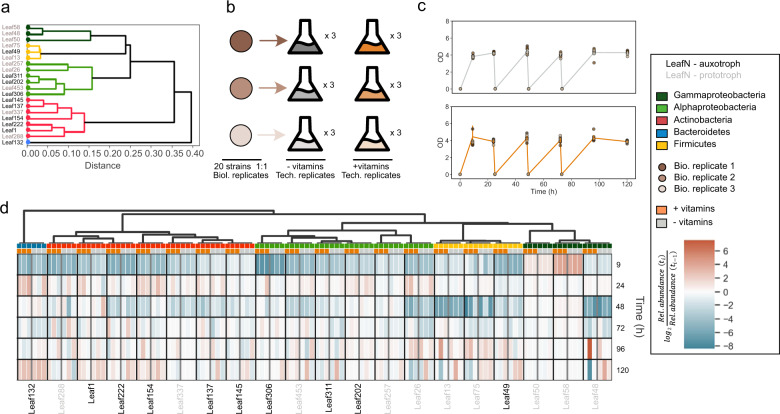
Co-culture experiment to determine whether auxotrophic strains are able to access vitamins from prototrophs. Source data in Source Data 6, 7. **a** Selection of strains for co-cultures. A total of 20 strains were chosen to represent the phylogenetic diversity in the *A. thaliana* leaf microbiota. Clustering is based on full-length 16S rRNA gene sequences. **b** Strains shown in panel A were mixed together in equal numbers (see Methods for details) in three biological inocula. Each inoculum was used to inoculate three technical replicates under two growth regimes: minimal medium with and without vitamins, resulting in 18 cultures. All 20 proteinogenic amino acids were added to all cultures (100 µM). **c** Growth measurements of the cultures from **b** Cultures were grown for a total of 120 h, and diluted at 24, 48, and 72 h. At 9, 24, 48, 72, 96, and 120 h (indicated as dots), samples for 16S rRNA gene sequencing (cells) and LC/MS (supernatants) were taken. **d** Relative abundance of all 20 strains (columns) in the co-culture experiment. For each strain, the average of the three technical replicates for each biological replicate is shown as the change of relative abundance compared to the previous time point, first in minimal medium with vitamins (columns labelled with orange boxes), then in minimal medium without vitamins (columns labelled with grey). For each strain, the three replicates in the same condition are separated by thin white lines and the two conditions are separated by thick gray lines. The 20 strains are separated by thick black lines. Strains are clustered by phylogeny as computed from 16S rRNA gene alignment.

Co-culture experiments can be used to provide insights into bacterial interactions in microbial communities [34–37]. Here, we asked whether auxotrophs access vitamins from co-cultivated prototrophs. To this end, we selected phylogenetically representative vitamin auxotrophic (*n* = 10) and prototrophic (*n* = 10) strains for a co-culture experiment (Fig. 4a). We conducted a serial dilution experiment of the strain mix (3 biological replicates each in 3 technical replicates) in minimal medium supplemented with 20 mM glucose and all 20 proteinogenic amino acids (100 μM each). The media differed only in whether they were supplemented with vitamins. (Fig. 4b, c). The cultures were analysed at regular intervals by 16S rRNA gene sequencing to determine the relative composition of the bacterial species. Growth in both conditions was comparable (Fig. 4c). We observed that the relative abundance profile between media was similar in both groups, i.e., auxotrophs and prototrophs, and independent of whether vitamins were supplemented or not (Fig. 4d, gray vs orange columns for each strain; Supplementary Fig. 8). The only auxotrophic species that benefited from supplemented vitamins was *Pedobacter* Leaf132 (*p-*value 0.003, rm-ANOVA). Two auxotrophic strains, *Rhizobium* Leaf202 and *Arthrobacter* Leaf137, had higher relative abundances in cultures where vitamins were not supplemented (*p* values <0.01 rm-ANOVA). This analysis thus shows that lack of external vitamin supplementation does not prevent the growth of auxotrophs when cultivated in a community with prototrophic strains and suggests vitamin cross-feeding.

**Fig. 5:**
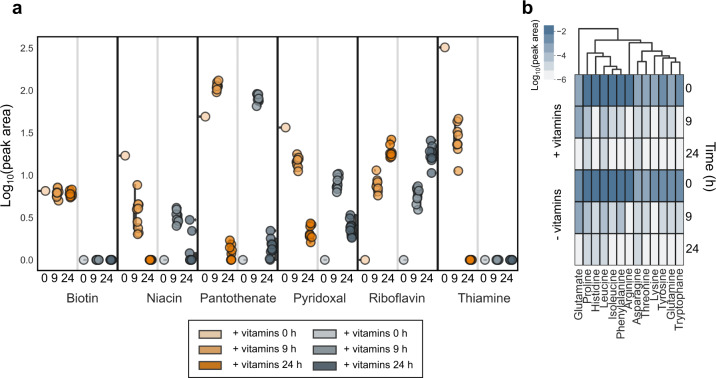
Exometabolomics for co-cultures. Source data in Source Data 8. **a** Time series of vitamin consumption in co-cultures as measured from co-culture supernatants. For each vitamin, the log_10_ of total ion current normalized peak area is first shown in vitamin-supplemented cultures, and then for vitamin-deplete cultures. **b** Amino acids measured from the co-culture supernatants. Data are log_10_ transformed peak areas normalized to the signal from medium which was supplemented with amino acids in both media (+ and –vitamins). Each cell in the heatmap represents the average of 9 replicates.

To assess the presence of cross-feeding, we examined the spent media from the co-cultures mentioned above (Fig. 5). We measured the media without bacteria (0 h time point) as well as supernatants after 9 h and 24 h of co-culture. We first compared the vitamin pools in the two conditions (± vitamins). When vitamins were supplemented, niacin, pantothenate, and thiamine concentration decreased between 0 h and 9 h, and further between 9 h and 24 h, suggesting net uptake by the bacteria grown in the medium (orange series in Fig. 5a). Decrease in the vitamin pools of these four vitamins was expected considering that half of the strains inoculated were auxotrophic for one or more of them. Biotin concentration, however, did not decrease during the experiment (orange series in the first panel in Fig. 5a) despite almost all of the strains being auxotrophic for it, indicating either small biotin uptake rates by auxotrophs, or that prototrophic strains secreted biotin at rates comparable to the uptake of biotin by auxotrophic strains. We also observed uptake of pyridoxal, for which no strain was auxotrophic. Riboflavin concentration was below the detection limit in the bacteria-free media, but it consistently accumulated in the medium during bacterial growth, suggesting﻿ that at least one of the strains in the co-culture secreted more riboflavin than others took up. In the vitamin-deplete medium, we found an accumulation of niacin, pantothenate, and pyridoxal at 9 h, followed by a decrease in these vitamins at 24 h, implying that the bacteria first secreted these vitamins into the medium and subsequently took them up (Fig. 5a, grey series). Thiamine and biotin pools remained below the detection limit. Amino acids that were supplemented in both media (alongside with glucose as the single main carbon source), decreased in all co-cultures when compared to the pure media, indicating net uptake from the medium (Fig. 5b). The majority of the provided glutamate (an attractive carbon and nitrogen source [38]) was still available after 9 h of co-culture and mainly consumed during the stationary phase.

### Auxotrophs benefit from co-cultures beyond vitamin supply

**Fig. 6:**
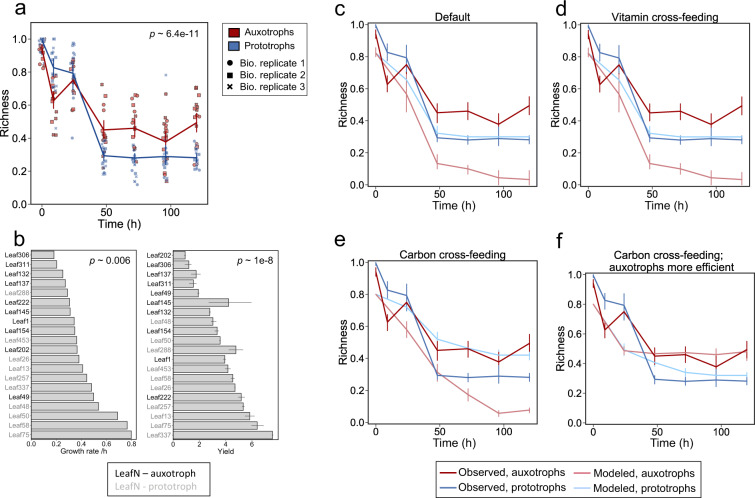
Auxotrophs benefit from co-cultures beyond vitamin supply. **a** Species richness in co-cultures cultivated in vitamin-supplemented medium. Source data in Source Data 6. **b** Experimentally determined growth rates and yields (=carrying capacity) for each strain growing in the vitamin-supplemented medium. Source data for growth rates in Source Data 9 and for yields in Source Data 10. **c–f** Species richness as modelled by consumer-resource models. Error bars for the models were obtained via sensitivity analyses as follows. In **c** and **d**, the threshold for a bacterium to count as “present” was varied from 0.1% to 1% of total abundance. In **e**, the threshold in growth rate for bacteria that were set to secrete the second carbon source was varied between 0.3 h^−1^ (*n* = 15) to 0.6 h^−1^ (*n* = 3). In **f**, the efficacy of auxotrophs to preferentially use the second carbon source was varied from 2 to 5 fold that of prototrophs’. Source data in Source Data 6. Source Data 6Source Data 9Source Data 10.

Auxotrophic mutants often have a fitness advantage over prototrophic ancestral strains in pairwise competition experiments [13, 24, 39]. In the following analysis, we assessed whether this also holds true in a community context of natural auxotrophs and prototrophs. Specifically, we predicted species richness from the growth rate and yield of each strain in individual cultures and compared these predictions to experimentally obtained data (Fig. 4). We conducted the analysis on relative abundance data in minimal medium supplemented with vitamins (orange columns in Fig. 4d) by comparing the species richness (number of detected species divided by the number of species originally introduced to the co-culture) over time in the two groups: auxotrophs and prototrophs. We found that two-thirds of the strains were undetected and hence interpreted as lost after the first two dilution cycles (after 24 h and 48 h) before eventually stabilizing at 72 h (Fig. 6a). More strains were lost in the prototrophic group: three-quarters of prototrophs were undetected after 48 h, significantly less than auxotrophs whereby only half were undetected (*p* ≪ 0.001, rm-ANOVA, Fig. 6a). To investigate whether the statistically different outcome between auxotrophs and prototrophs was to be expected, we attempted to predict species richness based on growth in monocultures. To this end, growth parameters were experimentally determined in the same vitamin-supplemented medium used for the co-culture experiments (Fig. 6b). Using these data, we parametrized a consumer-resource model and simulated the expected abundance of each strain in the vitamin-supplemented co-cultures. Briefly, the glucose concentration was iteratively updated to simulate eventual carbon limitation in a batch culture, growth rates were updated according to Monod kinetics, and no interaction between species was included (see Methods for modelling details). From the simulated abundances, we calculated the theoretical species richness. We found that in the prototrophic group, the simulation qualitatively captured the experimentally observed species richness (light blue line compared to the dark blue line in Fig. 6c), and correctly predicted the final species richness. All auxotrophic species were lost in the simulation (light red line compared to the dark red line in Fig. 6c), as expected due to their lower growth rates and yields compared to the prototrophic strains (Fig. 6b).

The apparent contradiction between the consumer-resource model and the experimental data prompted us to explore whether an extension of the models could capture the observed species richness. We began by implementing a vitamin cross-feeding term, which was not included in the initial model. From the exometabolomics measurements (Fig. 5), we learned that prototrophs secrete vitamins into the medium, thereby at least transiently increasing its vitamin concentration. Moreover, 16S rRNA gene sequencing of samples derived from cultures to which no vitamins were added indicated that the observed increase in vitamin concentration during co-cultivation with prototrophs was sufficient to support growth of the auxotrophs (Fig. 4). If the vitamin concentration in the growth medium became low enough to limit growth prior to carbon limitation, these extra vitamins could have resulted in an increase in yield. To this end, we acquired growth data in a range of physiologically relevant vitamin concentrations (Supplementary Fig. 9). The observed differences in both growth rate and yield were generally small. Nonetheless, we modified the growth of each strain in the consumer resource model from each strains’ growth parameters at 1 µM vitamins to those when grown in 10 µM vitamins, thereby reducing potential early growth arrest. We observed that this simulated increase in vitamin concentration did not result in increase of predicted auxotrophic species richness (Fig. 6d).

Consumer resource models with vitamin cross-feeding did not capture the experimentally observed difference in species richness (Fig. 6d). An alternative scenario for the high richness in the auxotrophic group of the co-culture is that they may utilize metabolic by-products as carbon sources. It is known that even under aerobic conditions, many fast-growing bacteria ferment their carbon source, resulting in secretion of by-products such a pyruvate, acetate, or succinate [40, 41]. Thus, changes in community composition during stationary phase (between 9 h and 24 h as well as 96 h and 120 h) is likely due to (co-)consumption of metabolic by-products after the main carbon source glucose is consumed. To address this scenario, we introduced a second carbon source, effectively mimicking secretion of a compound such as acetate by fast-growing prototrophic strains (Fig. 6b) into the model. We then allowed all slow-growing strains to grow on this newly available resource at rates proportional to their growth rate on glucose. As auxotrophs were enriched in the slow-growing group, they were able to preferentially use the by-product, while giving the prototrophic group an equal chance at growing on the secreted substrate given that the boundary condition of slow growth was met (see Supplementary Fig. 10 for details about concentration and effect to total cell number). Adding the carbon cross-feeding term indeed improved the predictions in comparison to the original model; both groups lose in species richness but neither group is entirely lost, and the species richness stabilises eventually (Fig. 6e). This model, however, still predicts the prototrophic group to have a higher final species richness. We, therefore, gave auxotrophs preferential access to the newly available carbon source by increasing their substrate affinity. In this scenario, the models captured the experimentally determined qualitative dynamics (Fig. 6f). Taken together, this analysis suggests that increased ability for carbon source cross-feeding determines the success of auxotrophic strains in co-cultures.

## Discussion

Coenzymes are not only ubiquitous but also generally conserved across all domains of life. They expand the catalytic toolbox of proteins and act as carrier molecules for organic acids and one-carbon units [1]. Here, we characterized the auxotrophic requirements of environmental bacteria isolated from the phyllosphere of *A. thaliana* and explored genomic and physiological traits that co-occur with the inability to synthesize vitamins. We found that vitamin auxotrophy was common for biotin, niacin, pantothenate, and thiamine and that auxotrophy was systematically associated with the absence of specific genes on the respective biosynthetic pathways. Although other studies have reported the absence of some of the genes identified in this study in other auxotrophs [7, 8], further studies will be required to assess whether auxotrophy is generally caused by the loss of these few specific genes. Furthermore, our experimental and bioinformatics analyses defined a set of auxotroph-specific functions as well as cross-feeding and storage of coenzymes in diverse naturally auxotrophic bacterial isolates.

After a strain loses a biosynthetic pathway, it becomes dependent on an external source of that nutrient. Therefore, its niche will be limited to those with at least occasional access to this resource. Hence, populations of strains may lose the ability to synthesize all compounds that the enforced niche can supply. Indeed, we observe multiple auxotrophies in four out of five confirmed auxotrophs (Fig. 1). Cross-feeding of nutrients was previously shown to promote coexistence [42]. In a two-strain system, a “beneficiary” strain (loss-of-synthesis mutant) did not outcompete the “helper” strain that supplied the nutrient. If a third strain that supplies the same nutrient was involved, the beneficiary outcompeted the helper strain; thus, one exchanged metabolite may only stabilize a community consisting of two strains. Communities found in nature may have hundreds or thousands of members. Therefore, multiple auxotrophies are required by each auxotrophic strain for cross-feeding interactions to stabilize large communities. As we observe multiple auxotrophies in 80% of auxotrophs, they may contribute to the observed stability of the community [30, 43] by cross-feeding. Therefore, we suggest that the identity of the required compound is dictated by the niche, but the number of auxotrophies per strain is rather an inherent property of the community. Although largely oligotrophic, there are detectable amounts of carbon sources (glucose, methanol, sucrose, fructose) [44], amino acids, and even vitamins on leaf surfaces, made available by either the plant host itself of the bacteria inhabiting the niche [6, 45, 46]. Auxotrophies for metabolically costly vitamin B12 (which is not used in plant metabolism) were not observed (Supplementary Fig. 11).

The genomes of bacterial populations change in response to selection pressure set by their environment and as random effects, e.g., genetic drift. For example, biosynthesis of diverse compounds requires allocation of cellular resources in terms of energy (mainly ATP and redox coenzymes) and protein synthesis, i.e., a cost. Provided that the product of such biosynthetic process is available in the environment, those organisms that lose the biosynthetic ability for that compound avoid the cost. In a number of studies, it was argued that by avoiding the cost of biosynthesis, cells gain a fitness advantage, which allows for selective advantage of loss of biosynthesis—this theory is called the Black Queen Hypothesis [4, 13, 39]. However, the loss of biosynthesis may also be neutral and caused by genetic drift. Smaller genomes are often observed in bacteria isolated from rich growth environments, and is strongly linked to endosymbiotic and parasitic lifestyles [32, 47–49]. It is however not possible to infer the mechanism between selection and genetic drift based on observed genome reduction.

Genome reduction can occur in two ways: 1. general genome reduction and 2. loss of specific genes [42]. In this study, we observed both specific and general absence of genes. However, most of the genome reduction fell under the first category as we observed up to 19% smaller genomes in auxotrophs, less than 1% of which can be explained by the lack of the genes directly related to auxotrophy (Figs. 2 and 3). We also observed genomic and metabolic reallocation, especially in niacin auxotrophs (Fig. 3b, c). Our analyses also indicate that auxotrophic bacteria have more enzymes that require the vitamin they are auxotrophic for (especially biotin and NAD) than prototrophs (Fig. 3b). This observation does not imply larger turnover or need for the coenzyme but rather how many reactions could be disturbed by limitation of the vitamin and therefore the impact that vitamin limitation would have on the metabolic network. Whether the requirement itself remains constant or even decreased depends on the extent to which the genes are translated. We hypothesize that the degree of dependency (here defined by the number of enzymes using the coenzyme) might not impact auxotrophs as much as it affects prototrophs because prototrophs might minimize their dependency on the coenzyme due to cost of vitamin biosynthesis.

The proportion of biosynthetic genes present in a pathway is often used to classify bacteria to auxotrophs or prototrophs [10, 13, 16]. Our results are in line with previously published work [18], suggesting that predicting auxotrophy based on the frequency of genes results in inaccurate predictions. We also observed that, although random gaps in genomes in general and coenzyme biosynthetic pathways in particular are frequently observed, lack of 1-3 specific coenzyme biosynthesis genes per pathway is  strongly associated with observed auxotrophy (Fig. 2**;** Table 1). Loss of some of the genes we identified here have already been associated with auxotrophy before [7, 8]. It remains an open question whether auxotrophy is universally caused by the loss of a few specific genes. Prediction accuracy of auxotrophies based on genomic data might thus be improved by, for example, giving a higher weight to genes that are known to be commonly lost in auxotrophs. In this study, we could predict auxotrophy with 80–100% accuracy, recall, and precision by training models with the selected features (Table 1).

We observed a higher robustness to biotin limitation in some auxotrophic strains (Fig. 1), which raises the question about the storage mechanism. In mammals, biotin storage has been linked to the mitochondrial acetyl-CoA carboxylase [50]. In biotin auxotrophic yeasts, histone biotinylation has been suggested to serve as potential biotin storage in auxotrophic *Candida albicans*, which is in contrast to biotin prototrophic yeast *Saccharomyces cerevisiae* that does not biotinylate its histones [51]. At its simplest, the storage could be unspecific and distributed among proteins that covalently bind biotin as the prosthetic group. This hypothesis is supported by the observation that biotin auxotrophs have more biotin-requiring enzymes in their metabolic networks (Fig. 3b). Whether elevated number of biotin-dependent enzymes is a storage strategy in bacteria remains to be tested, but is supported by a recent study that showed biotin-binding rhizavidin to serve in biotin storage for *Rhizobium* spp [52].

Auxotrophy arises frequently in evolution, and in this study, we identified auxotrophs in all major phyla within the *A. thaliana* leaf microbiota. Further, in this study (Figs. 4–6, Supplementary Fig. 8), we observe, in agreement with other studies, that auxotrophs persist even in vitamin-free co-cultures with prototrophs [12, 14, 16]. These observations invoke the question, whether auxotrophs have a designated role in microbial community assembly. Metabolic dissimilarity, at least theoretically and in the case of some amino acids, may drive cross-feeding [26]. Our results support this idea as we find auxotrophs and prototrophs to be metabolically and phylogenetically different from each other. In this study, we confirmed that auxotrophs maintain their population well in the absence of vitamins given that they are cultivated together with prototrophic strains that provide them vitamins. Therefore, we hypothesise that auxotrophs remain in growth arrest until enough vitamins are secreted to resume growth, and that this lifestyle shifts the selection pressure to efficient consumption of metabolic by-products released by other strains.

We found that auxotrophic strains were more successful in vitamin-supplemented co-cultures than expected from their growth in individual cultures. A consumer-resource model parametrized by experimentally obtained growth rate and yield data with a carbon cross-feeding term sufficiently captured this observation. As growth of auxotrophic strains cannot be resumed until prototrophs have secreted enough vitamins, preferentially using a carbon source secreted alongside with the vitamin could give auxotrophs a competitive edge. Our findings and inference are congruent with previous results where stable coexistence was explained by implementing a non-specific carbon cross-feeding term in a consumer-resource model framework, and that growth on such metabolic by-products is often comparable to growth on primary carbon source such as glucose [35]. Our framework suggests that not only does such metabolic facilitation promote coexistence, but preferential consumption of carbon by-products may also represent a viable strategy to avoid competitive exclusion. Taken together, our results suggest that auxotrophy is a part of a lifestyle that specializes in consumption of metabolic products of other bacteria and can therefore be beneficial for free-living bacteria that are a part of a microbial community.

## Materials and methods

### Strains and growth assays

All *At*-LSPHERE strains used in this study were previously published [30]. All growth assays were performed at 28 °C for *At*-LSPHERE strains and 37 °C for *E. coli* [53] strains. The turbidity of shake flask cultures was determined by measuring the optical density at 595 nm (=“OD_600_”) in semi-micro cuvettes (Bio-Greiner) using a Biophotometer Plus (Eppendorf). Samples were diluted as appropriate to keep the OD readings in the linear range. For batch cultures with continuous OD monitoring for 96 well plates (TPP flat bottom), Tecan Infinite M200 Pro was used at 1 mm amplitude orbital shaking.

### Media

The medium base was a phosphate buffer (2.4 g/l K_2_HPO_4_, 2.08 g/l NaH_2_PO_4_·2H_2_O) with mineral salts (1.62 g/l NH_4_Cl, 0.2 g/l MgSO_4_·7H_2_0). Glucose was added at 20 mM and pyruvate at 40 mM. For all media components, 10x stocks were prepared, dissolved in ddH_2_O and filter sterilized. Vitamins were supplemented when indicated in the following concentrations: D-pantothenic acid hemi calcium salt 1.05 µM, biotin 0.41 µM, riboflavin 1.06 µM, thiamine · HCl 1.19 µM, pyridoxal · HCl 0.98 µM, p-amino benzoic acid 1.09 µM, cobalamine 0.14 µM, lipoic acid 0.24 µM, niacin 1.22 µM, folic acid 0.23 µM. All 20 proteinogenic L-amino acids were supplemented at 0.1 mM each, except L-tryptophan and L-tyrosine were supplemented at 0.05 mM. 1000x stocks were prepared for all other vitamins and amino acids except for riboflavin and tryptophan for which 100x stocks were prepared.

### Growth analysis

Linear regression on ln transformed data was carried out using linear_regression function from sklearn. Datapoints that lied outside of log-linear scale were omitted. Growth rates were only calculated for cultures that performed at least 2 doublings in exponential phase.

In depletion experiments (see Fig. 1 and Supplementary Fig. 2), growth of cells cultivated on vitamin-free media was defined to be deviating from the supplemented control if the following criteria were fulfilled. There had to be at least a 0.25 doubling difference in the total number of doublings in the vitamin-free and vitamin-supplemented cultures. Additionally, in at least two out of the three replicates the growth rate after the 0.25 doubling separation had to be reduced by 25%; in the remaining replicate, a 10% reduction was considered acceptable. The storage was defined as the number of doublings a strain performed without the vitamin at this time point. Solid R2A medium was obtained from Sigma.

### Auxotrophy screens

In order to screen all 224 bacteria in the *At*-LSPHERE collection [30] for growth in 4 media (+vitamins and amino acids, −supplements, +amino acids and +vitamins), each strain was inoculated in liquid R2A from a solid R2A plate and let grow to stationary phase (24 h). Then, 10 µl of stationary-phase pre-culture was inoculated in 190 µl of each of the 4 media for an overnight pre-culture at 28 °C on a 220 rpm shaker in three biological replicates. Main cultures were inoculated in the same fashion, and OD measurements were taken at two time points: *t* = 0 and *t* = 24 h. The OD was normalized to the OD in control (cell-free, medium-filled) wells. For the drop-out screen with 50 strains, same protocol was repeated with a few changes. Here, 5 µl of stationary-phase pre-culture on R2A was inoculated in 45 µl of each of the 4 media (+vitamin and amino acids, −supplements, +vitamins, +amino acids) and each of the 30 drop-out media (each individual compound in Source Data 1) for the overnight pre-culture and again for the main culture. Every 24 h, a 5 µl of stationary-phase culture was transferred to solid R2A plates and colony color and morphology were compared to known characteristics of each strain to exclude contaminations.

### High-throughput depletion experiments

Each strain was inoculated from solid R2A plates on a 96 well plate in biological triplicates and five technical replicates in a medium supplemented with 20 amino acids and 4 vitamins: thiamine, niacin, biotin, and pantothenate at 200 µl culture volume. Main cultures were prepared next day by diluting 10 µl of each cell culture into fresh media. After an overnight culture, late exponential stage was reached. The five technical replicates were pooled together into a sterile 2 ml Eppendorf tube and washed twice by centrifuging the cell suspension tube (10,000 rpm, 2 min), removing supernatant, and dissolving the cell pellet in 1.5 ml of sterile MgCl_2_. After the second wash, cells were collected via a centrifugation step as before, and the resulting pellets were dissolved in 50 µl of MgCl_2_. 5 µl of the resulting cell suspension was inoculated in 195 µl of five media that contained all 20 amino acids and differed in vitamin supplements. One of the media was a control medium, and it included all four vitamins as the preculture medium. The other four media each lacked one of the following vitamins: thiamine, biotin, niacin, or pantothenate. Growth was then monitored on a Tecan plate reader (for details see “Growth assays”). After every 2 doublings, cultures were diluted as appropriate to maintain exponential growth in the plate reader’s linear range.

### Sample preparation for LC/MS analysis of depletion experiments

*Rhizobium* Leaf68 was cultivated in 20 ml culture in a medium containing 20 amino acids and biotin, niacin, and pantothenate. In late-exponential phase (OD~1), cells were collected via centrifugation (10,000 rpm, 2 minutes), followed by two rounds of washing and pelleting with 20 ml of sterile MgCl_2_ and 10,000 rpm for 2 min. Afterwards, cells were collected via centrifugation and dissolved in 1 ml of MgCl_2_. Cultures were inoculated in four shake flask cultures with 100 µl of this inoculum: one of the cultures was supplemented with all 3 vitamins, and a drop-out medium for each vitamin, respectively. OD was monitored once per doubling, and the intracellular metabolome was sampled as follows. Cultures were kept at 28 °C in a shaking water bath, and sample volume was determined based on the OD (1/OD ml). The determined volume of cell suspension was pipetted onto a filter standing atop a suction flask to remove the medium. Cells standing on filter were washed with 10 ml of dH_2_O that was kept at 28 °C in water bath, and the washed filter was subsequently transferred to a Schott flask containing 8 ml of cold acidified (−20 °C) quenching solution (3 parts MeCN:1 part MeOH:1 part 0.05 M formic acid). After 10 minutes, the quenching solution was transferred to a 50 ml Falcon tube and lyophilized at −50 °C overnight. The resulting powder was dissolved in 250 µl of pre-cooled dH_2_O, and kept in −80 °C until analysis.

### LC/MS analysis

LC separation was achieved with a Thermo Ultimate 3000 UHPLC system (Thermo Scientific) at a flow rate of 500 µl min^–1^. Two different separation methods were applied. First separation was achieved by hydrophilic interaction (HILIC; Aquity UHPLC BEH Amide column [100 × 2.1 mm, 1.7 µm particle sizes; Waters]) as described in [54]. For HILIC analysis, 50 µl of the aqueous sample was dried (SpeedVac) and dissolved in MeCN. The C18 reversed phase (C18RP) separation was achieved using a Kinetex XB-C18 column (particle size 1.7 µm, pore size 100 Å; dimensions 50 × 2.1 mm^2^, Phenomenex) as described elsewhere [55]. For mass analysis, LC instrument was coupled to a Thermo QExactive plus instrument (Thermo Fisher Scientific), and the mass spectrometer was operated both positive and negative FTMS mode at mass resolution of 30,000 (*m/z* = 400). Heated electro spray ionization (ESI) probe was used applying the following source parameters: vaporizer 350 °C; aux gas 5; ion spray voltage +3.5 kV, sheath gas, 50; sweep gas, 0; radio frequency level, 50.0; capillary temperature, 275 °C. To analyze the data, targeted extraction of ion chromatograms was conducted using emzed2 [56]. Retention time windows were determined based on chemical standards, and selected windows were normalized to background signal.

### Co-culture experiments

As a pre-experiment, CFU counts per unit of OD were determined using a dilution plating method. Colonies were counted from a dilution that allowed for determination from 5 µl dots (Supplementary Table 5). All 20 strains were then inoculated from solid R2A plates into liquid medium containing 20 amino acids and 10 vitamins and grown overnight in biological triplicates in 10 ml culture volume. After the pre-culture, all strains were in stationary phase. The OD of each culture was measured and adjusted to 5e8 cells per ml. These adjusted cultures were then combined into an inoculum where each strain had an abundance of approximately 1/20. The inoculum was washed as described in section “Sample preparation for LC/MS analysis of depletion experiments”. Five sequencing samples were taken from each inoculum, and three technical replicates in both vitamin-supplemented and vitamin-free media were inoculated from each inoculum. Culture volumes were 20 ml, and cultures were allowed to grow at 28 °C with 200 rpm orbital shaking. At 9, 24, 48, 72, 96, and 120 h, samples for 16S rRNA gene sequencing and exometabolomics were taken as follows. For 16S rRNA gene sequencing, a 1 ml sample was transferred to a DNA extraction tube from FastDNA SPIN Kit for Soil. The tubes were centrifugated (10,000 rpm, 5 minutes, 4 °C), supernatants were removed, and the tubes with cell pellets were frozen and kept at -80 °C until extraction. For exometabolomics, 1 ml of culture was transferred to a 2 ml Eppendorf tube and centrifugated (10,000 rpm, 5 min, 4 °C). 200 µl of supernatants were stored in two technical replicates on a 96 well plate and stored at −20 °C until analysis (see “LC/MS analysis” for details).

### 16S rRNA gene amplicon library preparation and sequencing

DNA was extracted using the FastDNA SPIN Kit for Soil (MP Biomedicals) following the manufacturer’s instructions. The samples were transferred to DNA low-binding 96-well plates (Frame Star 96, semiskirted), the DNA concentration was quantified using double-stranded DNA QuantiFluor (Promega) and normalized to 1 ng µl^−1^. The 16S rRNA gene amplicon library was generated as follows. PCR amplification, clean-up, and barcoding PCR were performed as in [57]. DNA concentration was determined as above, and each well was normalized to 1 ng µl^−1^. Equal volume from each well was then combined into a pooled 16S rRNA gene amplicon library, and the library was cleaned twice with AMPure magnetic beads using a bead-to-DNA ratio of 0.9 to remove small DNA fragments. The amplicon length distribution of the library was assessed on a 2200 TapeStation using HS D1000 (Agilent), resulting in an effective library size of 554–643 bp. Sequencing was performed for 12 pM samples on a MiSeq desktop sequencer (Illumina) at the Genetic Diversity Centre Zurich using the MiSeq reagent kit v.3 (paired end, 2 × 300 bp, 600 cyc PE). Denaturation, dilution, and addition of 15% PhiX to the DNA library were performed according to the manufacturer’s instructions. Custom sequencing primers were used as described previously [30].

### Comparative genomics analyses

The genome of each strain was obtained querying RefSeq with the accession numbers in Source Data 5. Genes were mapped to COG terms using eggnog mapper and all strains were then labelled as either auxotroph or prototroph iteratively for each of the following compounds: biotin, thiamine, pantothenate, niacin. The analysis was restricted to COG terms for which a pathway mapping is provided (https://www.ncbi.nlm.nih.gov/research/cog/pathways/). A contingency table was then built based on the presence of each COG term in auxotrophs and prototrophs respectively. A χ^2^ test was performed on the contingency table, and *p*-values as well as information about which group had the higher presence of each COG term was stored. Resulting *p* values were subsequently corrected using the Benjamini-Hochberg method. For significantly different (*q*-value < 0.05) COG terms, a functional annotation was retrieved from COG database API (https://www.ncbi.nlm.nih.gov/research/cog/api/cog/) and mapped to biological process via KEGG (available from Biopython). The effectiveness of multiple testing correction was confirmed by generating random models by shuffling the auxotrophy/prototrophy labels. No COG term was significantly differently abundant between randomly assigned auxotrophs and prototrophs.

To create models for predicting auxotrophy from COG annotations, we trained decision tree classifiers based on the feature selection process presented above, or randomly chosen COG terms from each pathway (500 randomizations). First, we appended the dataset of 35 validated strains with additional 28 strains from the drop-out screen presented in Supplementary Fig. 1 to decrease sampling bias. This total set of 63 was divided to balanced training and testing datasets using 40% of the data for testing using “train_test_split” and decision tree classifiers were generated with “DecisionTreeClassifier” from sklearn library with a maximum depth of three nodes.

### Consumer-resource models

Consumer-resource models were applied to analyze the success of auxotrophs in co-culture experiments. Consumer-resource models are representations of an organism’s abundance as a function of its ability to consume a given resource. Here, we applied a consumer-resource model to determine the abundance of each bacterial strain using its experimentally observed CFU count (Supplementary Table 5), growth rate (μ), and yield. The carrying capacity **C** for each strain was determined by multiplying its experimentally-determined maximum yield by the CFU count per OD (CFU/OD column in Supplementary Table 5). All parameters are clarified in Supplementary Table 6.

### Default model

At *t* = 0, each strain’s abundance (CFU/ml) was set to its experimentally-observed abundance in the beginning of the experiment (the CFU/ml column in Supplementary Table 7) divided by 4 (as the strains were first mixed together, diluting each strain’s abundance by a factor of 20, the inoculum was inoculated in 1/200 into fresh medium, and finally multiplied by 1000 to estimate the CFU/ml).

The concentration of each strain was then updated for each time point. Here, the growth rate of the strain was first updated according to Monod kinetics

$$\mu _{Strain} = \mu _{Strain,\,\max } \ast \frac{{\left[ S \right]}}{{\left[ S \right] + K_S}}$$

Where substrate affinity K_s_ was assumed to be proportional to the strain’s yield (in terms of OD) and calculated by dividing the maximum yield of all strains by each strain’s yield. For each strain, an ordinary differential equation was formulated to describe change in that strain’s concentration:

$$\frac{{d\left[ {Strain} \right]}}{{dt}} = \mu _{Strain} \ast \left[ {Strain} \right] \ast \frac{{1 - \left[ {Strain} \right]}}{{C_{Strain}}}$$

Where C_strain_ is the carrying capacity or yield of that strain. These strain-specific equations were coupled to an ODE describing change in glucose concentration where [Glucose] was initially set to 20 mM.

$$\frac{{d\left[ {Glucose} \right]}}{{dt}} = sum\left( {\frac{{10 \ast \overline {\mu _{Strain}} \ast \overline {[Strain]} }}{{\overline {C_{Strain}} }}} \right) \ast \left[ {Glucose} \right]$$

Where the first part under the sum estimates each’s strains glucose consumption rate from its growth parameters (growth rate multiplied by 10). The total glucose consumption is then estimated from the consumption rates and number of cells scaled to carrying capacity. The unit under the sum is h^−1^. The model was then solved using odeint solver from scipy.

At *t*∈{24,48,72,96} a dilution step (1/200) was simulated by the following:

$$\overline {\left[ {Strain} \right]} = \overline {[Strain]} /200$$

$$\left[ S \right] = 20$$

$$\mu _{strain} = \mu _{strain,\,\max }$$

### Extension 1: Vitamin cross-feeding

The carrying capacity **C** was determined for each strain separately as described above. Based on data presented in Fig. 5, we estimated that vitamin concentration increased maximally 10-fold in the presence of prototrophic strains. Since 1 µM vitamins were supplemented to cultures, we repeated the simulation described above with the carrying capacity *C*_*Strain*_ for each strain based on their experimentally determined yield with 10 µM vitamins (Supplementary Fig. 9). For strains with lacking data, the average increase in carrying capacity was used instead.

### Extension 2: Carbon cross-feeding

The second carbon source was simulated by setting a secretion flux only for strains whose growth rate was greater than a given threshold (varied from 0.3 ^−h^ to 0.6 h^−1^). The secretion flux was scaled down by 50% from the glucose uptake rate. At *t* = 0, S_2_ = 0.

The secretion of S_2_ into the medium by fast-growing strains was therefore controlled by

$$r_{S_2} = \left\{ {\begin{array}{*{20}{c}} {r_{glc,\,strain} \ast 0.5,}&\mu _{strain} \ge threshold \\ \hfill0,&{\mu _{strain} \, < \, threshold} \end{array}} \right.$$

The change in concentration of [S_2_] was then

$$\frac{{d\left[ {S_2} \right]}}{{dt}} = sum\left( {\frac{{10 \ast \overline {r_{s2,\,Strain}} \ast \overline {\left[ {Strain} \right]} }}{{\overline {C_{Strain}} }}} \right) \ast \left[ {S_2} \right]$$

The formula for concentration and production rate of S_2_ led to biologically realistic concentration range (~5 mM; Supplementary Fig. 10). On the receiving end, the growth rates for S_2_ were estimated as follows

$$\mu _{2,\,Strain} = \mu _{Strain,\,\max } \ast Scaling\_factor \ast \frac{{\left[ {S_2} \right]}}{{\left[ {S_2} \right] + K_{S_2}}}$$

For simulations in which the auxotrophs and prototrophs were equally efficient, the *Scaling_factor* parameter was set to 1/3. In order to make auxotrophs more efficient, the scaling factor was set to 1 for auxotrophs and kept at 1/3 for prototrophs. The sensitivity for this parameter was tested by setting it in range from 1/2 to 2. The concentration of each strain was simulated as follows:

$$\frac{{d\left[ {Strain} \right]}}{{dt}} = \mu _{Strain,\,glu\cos e} \ast \left[ {Strain} \right] \ast \frac{{1 - \left[ {Strain} \right]}}{{C_{Strain}}} + \mu _{2,\,Strain,} \ast \left[ {Strain} \right] \ast \frac{{1 - \left[ {Strain} \right]}}{{C_{S2,\,Strain}}}$$

Where $C_{S_{2,\,Strain}}$was set to 70. This parameter was set based on the finding that growth on metabolic by-products can be comparable to growth on glucose [49].

The simulations were repeated for time *t*∈{1,2,3,…,120}. At *t*∈{24,48,72,96}, dilution was simulated as described above.

### Analysis software and statistical analysis

Unless otherwise stated, all analyses were performed on a Windows machine running Python 3.8 via Anaconda3 using custom scripts. Data were handled in pandas dataframes (V 1.1.3), for numerical computing numpy library (V 1.20.1) was used, and linear regression and multiple testing correction were performed via the sklearn and statsmodels (V 0.23.0 and V 0.12.0, respectively) implementations. For statistical testing, scipy (V 1.5.2) implementations were used. API’s were queried via requests (V 2.22.0) and KEGG via Biopython (V 1.76). Metabolic models were generated using CarveMe [33] (V 1.2.2) following the published tutorial (https://carveme.readthedocs.io/en/latest/usage.html). For continuous data, *t-*test or Welch’s *t* test was performed depending on variance within each group. For categorical data, χ^2^ tests were conducted. The number of replicates (*n*) and the type of test conducted can be found in respective Figure caption.

## Supplementary information

Supplementary Material

## Data Availability

Relevant code used to generate consumer resource models are available as Supplementary material. Raw LC/MS metabolomics data for supernatants and depletion experiments are available in MetaboLights https://www.ebi.ac.uk/metabolights/index (Accession ID MTBLS5309). Raw sequencing data are available in European Nucleotide Archive https://www.ebi.ac.uk/ena/browser/home?show=reads (Accession ID PRJEB55397).

## Source data

Source Data
Supplementary Tables
Python Consumer resource models

## References

[1] Fischer JD, Holliday GL, Rahman SA, Thornton JM (2010). The structures and physicochemical properties of organic cofactors in biocatalysis. J Mol Biol.

[2] Hartl J, Kiefer P, Meyer F, Vorholt JA (2017). Longevity of major coenzymes allows minimal *de novo* synthesis in microorganisms. Nat Microbiol.

[3] Nielsen J (2017). Metabolism: Built on stable catalysts. Nat Microbiol.

[4] D’Souza G, Kost C (2016). Experimental evolution of metabolic dependency in bacteria. PLOS Genet.

[5] Hockney RC, Scott TA (1979). The isolation and characterization of three types of vitamin R6 auxotrophs of *Escherichia coli* K12. J Gen Microbiol.

[6] Yoshida Y, Iguchi H, Sakai Y, Yurimoto H (2019). Pantothenate auxotrophy of *Methylobacterium* spp. isolated from living plants. Biosci Biotechnol Biochem.

[7] Bouvet O, Bourdelier E, Glodt J, Clermont O, Denamur E (2017). Diversity of the auxotrophic requirements in natural isolates of *Escherichia coli*. Microbiol.

[8] Seif Y, Choudhary KS, Hefner Y, Anand A, Yang L, Palsson BO (2020). Metabolic and genetic basis for auxotrophies in Gram-negative species. Proc Natl Acad Sci USA.

[9] Zarecki R, Oberhardt MA, Reshef L, Gophna U, Ruppin E (2014). A novel nutritional predictor links microbial fastidiousness with lowered ubiquity, growth rate, and cooperativeness. PLoS Comput Biol.

[10] Magnúsdóttir S, Ravcheev D, De Crécy-Lagard V, Thiele I (2015). Systematic genome assessment of B-vitamin biosynthesis suggests cooperation among gut microbes. Front Genet.

[11] Rodionov DA, Arzamasov AA, Khoroshkin MS, Iablokov SN, Leyn SA, Peterson SN (2019). Micronutrient requirements and sharing capabilities of the human gut microbiome. Front Microbiol.

[12] Sharma V, Rodionov DA, Leyn SA, Tran D, Iablokov SN, Ding H (2019). B-Vitamin sharing promotes stability of gut microbial communities. Front Microbiol.

[13] D’Souza G, Waschina S, Pande S, Bohl K, Kaleta C, Kost C (2014). Less is more: selective advantages can explain the prevalent loss of biosynthetic genes in bacteria. Evolution..

[14] Romine MF, Rodionov DA, Maezato Y, Osterman AL, Nelson WC (2017). Underlying mechanisms for syntrophic metabolism of essential enzyme cofactors in microbial communities. ISME J.

[15] Rodionova IA, Li X, Plymale AE, Motamedchaboki K, Konopka AE, Romine MF (2015). Genomic distribution of B-vitamin auxotrophy and uptake transporters in environmental bacteria from the Chloroflexi phylum. Environ Microbiol Rep.

[16] Soto-Martin EC, Warnke I, Farquharson FM, Christodolou M, Horgan G, Derrien M (2020). Vitamin biosynthesis by human gut butyrate-producing bacteria and cross-feeding in synthetic microbial communities. mBio..

[17] Garcia SL, Buck M, McMahon KD, Grossart H-P, Eiler A, Warnecke F (2015). Auxotrophy and intrapopulation complementary in the ‘interactome’ of a cultivated freshwater model community. Mol Ecol.

[18] Price MN, Zane GM, Kuehl JV, Melnyk RA, Wall JD, Deutschbauer AM (2018). Filling gaps in bacterial amino acid biosynthesis pathways with high-throughput genetics. PLOS Genet.

[19] Wilson AC, Pardee AB (1962). Regulation of flavin synthesis by *Escherichia coli*. J Gen Microbiol.

[20] Wilkinson JF (1963). Carbon and energy storage in bacteria. J Gen Microbiol.

[21] Kulakovskaya T (2014). Phosphorus storage in microorganisms: Diversity and evolutionary insight. Biochem Physiol Open Access.

[22] Brauer MJ, Yuan J, Bennett BD, Lu W, Kimball E, Botstein D (2006). Conservation of the metabolomic response to starvation across two divergent microbes. Proc Natl Acad Sci USA.

[23] Mason-Jones K, Robinson SL, Veen GF, Manzoni S, van der Putten WH (2021). Microbial storage and its implications for soil ecology. ISME J.

[24] Zamenhof S, Eichhorn HH (1967). Study of microbial evolution through loss of biosynthetic functions: Establishment of “defective” mutants. Nature..

[25] Mee MT, Collins JJ, Church GM, Wang HH (2014). Syntrophic exchange in synthetic microbial communities. Proc Natl Acad Sci USA.

[26] Giri S, Oña L, Waschina S, Shitut S, Yousif G, Kaleta C (2021). Metabolic dissimilarity determines the establishment of cross-feeding interactions in bacteria. Curr Biol.

[27] de Crécy-Lagard V, Haas D, Hanson AD (2018). Newly-discovered enzymes that function in metabolite damage-control. Curr Opin Chem Biol.

[28] Linster CL, Van Schaftingen E, Hanson AD (2013). Metabolite damage and its repair or pre-emption. Nat Chem Biol.

[29] Mülleder M, Capuano F, Pir P, Christen S, Sauer U, Oliver SG (2012). A prototrophic deletion mutant collection for yeast metabolomics and systems biology. Nat Biotechnol.

[30] Bai Y, Müller DB, Srinivas G, Garrido-Oter R, Pothoff E, Rott M (2015). Functional overlap of the *Arabidopsis* leaf and root microbiota. Nature..

[31] Hubalek V, Buck M, Tan B, Foght J, Wendeberg A, Berry D (2017). Vitamin and amino acid auxotrophy in anaerobic consortia operating under methanogenic conditions. mSystems..

[32] Morris JJ, Lenski RE, Zinser ER (2012). The black queen hypothesis: Evolution of dependencies through adaptive gene loss. mBio..

[33] Machado D, Andrejev S, Tramontano M, Patil KR (2018). Fast automated reconstruction of genome-scale metabolic models for microbial species and communities. Nucleic Acids Res.

[34] Foster KR, Bell T (2012). Competition, not cooperation, dominates interactions among culturable microbial species. Curr Biol.

[35] Goldford JE, Lu N, Bajić D, Estrela S, Tikhonov M, Sanchez-Gorostiaga A (2018). Emergent simplicity in microbial community assembly. Science.

[36] Blasche S, Kim Y, Mars RAT, Machado D, Maansson M, Kafkia E (2021). Metabolic cooperation and spatiotemporal niche partitioning in a kefir microbial community. Nat Microbiol.

[37] Pacheco AR, Osborne ML, Segrè D (2021). Non-additive microbial community responses to environmental complexity. Nat Commun.

[38] Zampieri M, Hörl M, Hotz F, Müller NF, Sauer U (2019). Regulatory mechanisms underlying coordination of amino acid and glucose catabolism in *Escherichia coli*. Nat Commun.

[39] D’Souza G, Waschina S, Kaleta C, Kost C (2015). Plasticity and epistasis strongly affect bacterial fitness after losing multiple metabolic genes. Evolution..

[40] Basan M (2018). Resource allocation and metabolism: the search for governing principles. Curr Opin Microbiol.

[41] Basan M, Hui S, Okano H, Zhang Z, Shen Y, Williamson JR (2015). Overflow metabolism in *E. coli* results from efficient proteome allocation. Nature..

[42] Mas A, Jamshidi S, Lagadeuc Y, Eveillard D, Vandenkoornhuyse P (2016). Beyond the Black Queen Hypothesis. ISME J.

[43] Carlström CI, Field CM, Bortfeld-Miller M, Müller B, Sunagawa S, Vorholt JA (2019). Synthetic microbiota reveal priority effects and keystone strains in the *Arabidopsis* phyllosphere. Nat Ecol Evol.

[44] Ryffel F, Helfrich EJ, Kiefer P, Peyriga L, Portais JC, Piel J (2015). Metabolic footprint of epiphytic bacteria on *Arabidopsis thaliana* leaves. ISME J.

[45] Tukey HB (1970). The leaching of substances from plants. Annu Rev Plant Physiol.

[46] Dong W, Stockwell VO, Goyer A (2015). Enhancement of thiamin content in *Arabidopsis thaliana* by metabolic engineering. Plant Cell Physiol.

[47] McCutcheon JP, Moran NA (2007). Parallel genomic evolution and metabolic interdependence in an ancient symbiosis. Proc Natl Acad Sci USA.

[48] Gil R, Sabater-Muñoz B, Latorre A, Silva FJ, Moya A (2002). Extreme genome reduction in *Buchnera* spp.: Toward the minimal genome needed for symbiotic life. Proc Natl Acad Sci U S A.

[49] Moran NA (2002). Microbial minimalism: Genome reduction in bacterial pathogens. Cell..

[50] Shriver BJ, Roman-Shriver C, Allred JB (1993). Depletion and repletion of biotinyl enzymes in liver of biotin-deficient rats: Evidence of a biotin storage system. J Nutr.

[51] Hasim S, Tati S, Madayiputhiya N, Nandakumar R, Nickerson KW (2013). Histone biotinylation in *Candida albicans*. FEMS Yeast Res.

[52] Vargas-Lagunas C, Mora Y, Aguilar A, Reyes-Gonzalez AR, Arteage-Ide A, Dunn MF (2022). A Tar aspartate receptor and Rubisco-like protein substitute biotin in the growth of rhizobial strains. Microbiol.

[53] Baba T, Ara T, Hasegawa M, Takai Y, Okumura Y, Baba M, et al. Construction of *Escherichia coli* K-12 in-frame, single-gene knockout mutants: The Keio collection. Mol Syst Biol. 2006;2:2006.0008. 10.1038/msb4100050 PMC1681482 16738554

[54] Mülleder M, Bluemlein K, Ralser M (2017). A high-throughput method for the quantitative determination of free amino acids in *Saccharomyces cerevisiae* by hydrophilic interaction chromatography-tandem mass spectrometry. Cold Spring Harb Protoc.

[55] Peyraud R, Kiefer P, Christen P, Massou S, Portais JC, Vorholt JA (2009). Demonstration of the ethylmalonyl-CoA pathway by using ^13^C metabolomics. Proc Natl Acad Sci USA.

[56] Kiefer P, Schmitt U, Vorholt JA (2013). EMZed: An open source framework in Python for rapid and interactive development of LC/MS data analysis workflows. Bioinformatics..

[57] Pfeilmeier S, Petti GC, Bortfeld-Miller M, Daniel B, Field CM, Sunagawa S (2021). The plant NADPH oxidase RBOHD is required for microbiota homeostasis in leaves. Nat Microbiol.

