# Supplementary material for: Metabolic adaptation to vitamin auxotrophy by leaf-associated bacteria
Source: ISME J. 2022 Aug 20;16(12):2712–24. doi: 10.1038/s41396-022-01303-x (PMC9666465; doi:10.1038/s41396-022-01303-x)
Supplement: Supplementary file 1 — Supplementary Material [file 41396_2022_1303_MOESM1_ESM.docx]

## Supplementary Figures


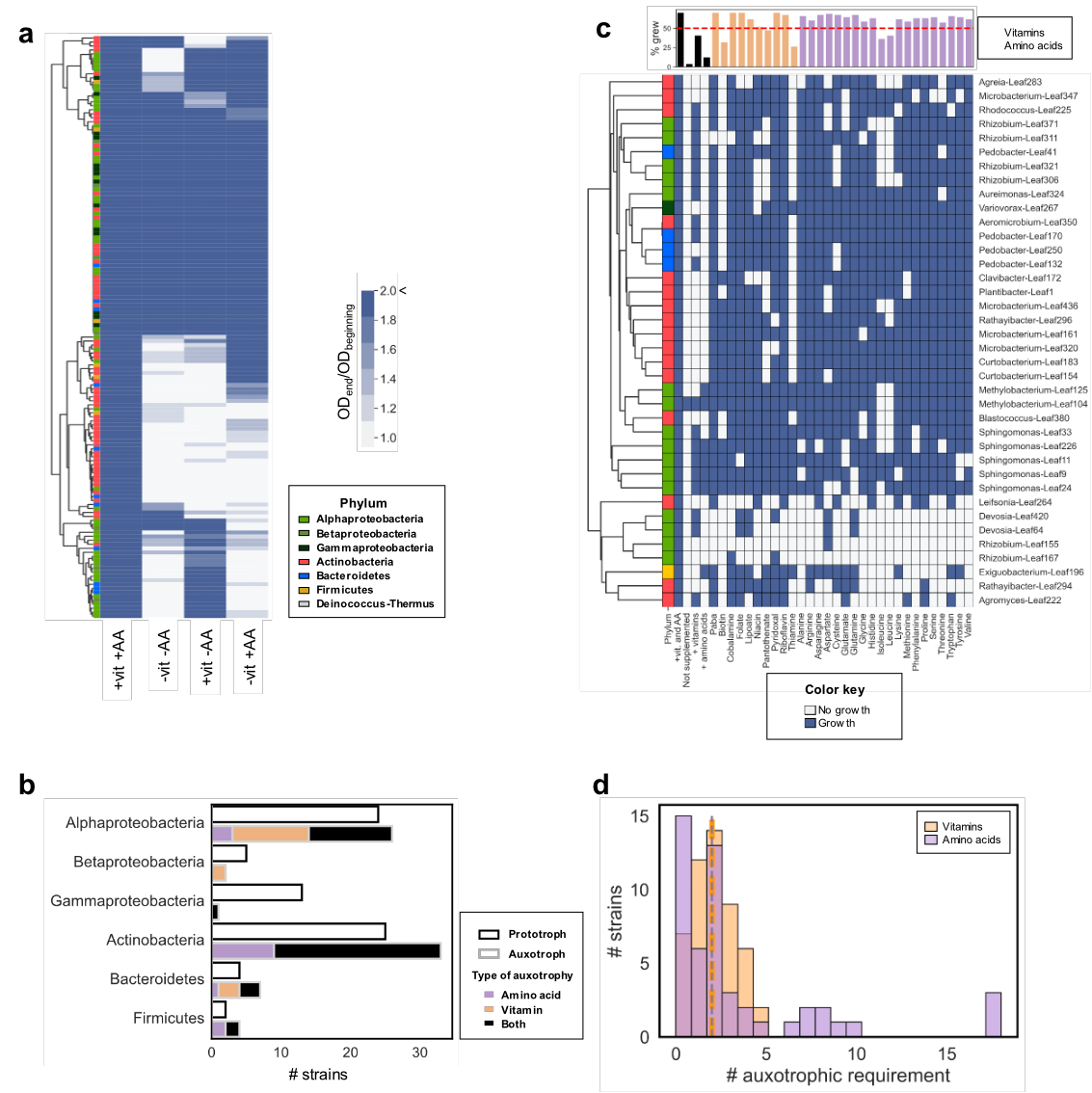


**Supplementary Fig. 1 Identifying auxotrophic strains and characterizing auxotrophy in *Arabidopsis thaliana* leaf microbiota.** Source data in **Supplementary Source Data 1**. **a)** Growth of each strain (rows) in minimal media with one of the following supplement regimes: +vit +AA = with vitamins and amino acids, -vit -AA = without vitamins and without amino acids, +vit -AA = with vitamins, without amino acids, or -vit +AA = without vitamins, with amino acids. The darkest color indicates that the OD in the main culture increased at least two-fold and is classified as growth. Rows are clustered by heatmap values. **b)** Phylum distribution of putatively auxotrophic and prototrophic strains**.** For auxotrophs, overlap between vitamin and amino acid auxotrophy is shown in stacked bars. **c)** Growth in individual vitamin and amino acid drop-out media. Data are shown for 50 strains (rows), and rows are clustered by heatmap values. **d)** Frequency of auxotrophic requirements (vitamins+amino acids). The median in both distributions are marked with the dashed lines (orange for vitamins and purple for amino acids). Data are computed for the 50 strains shown in **c**


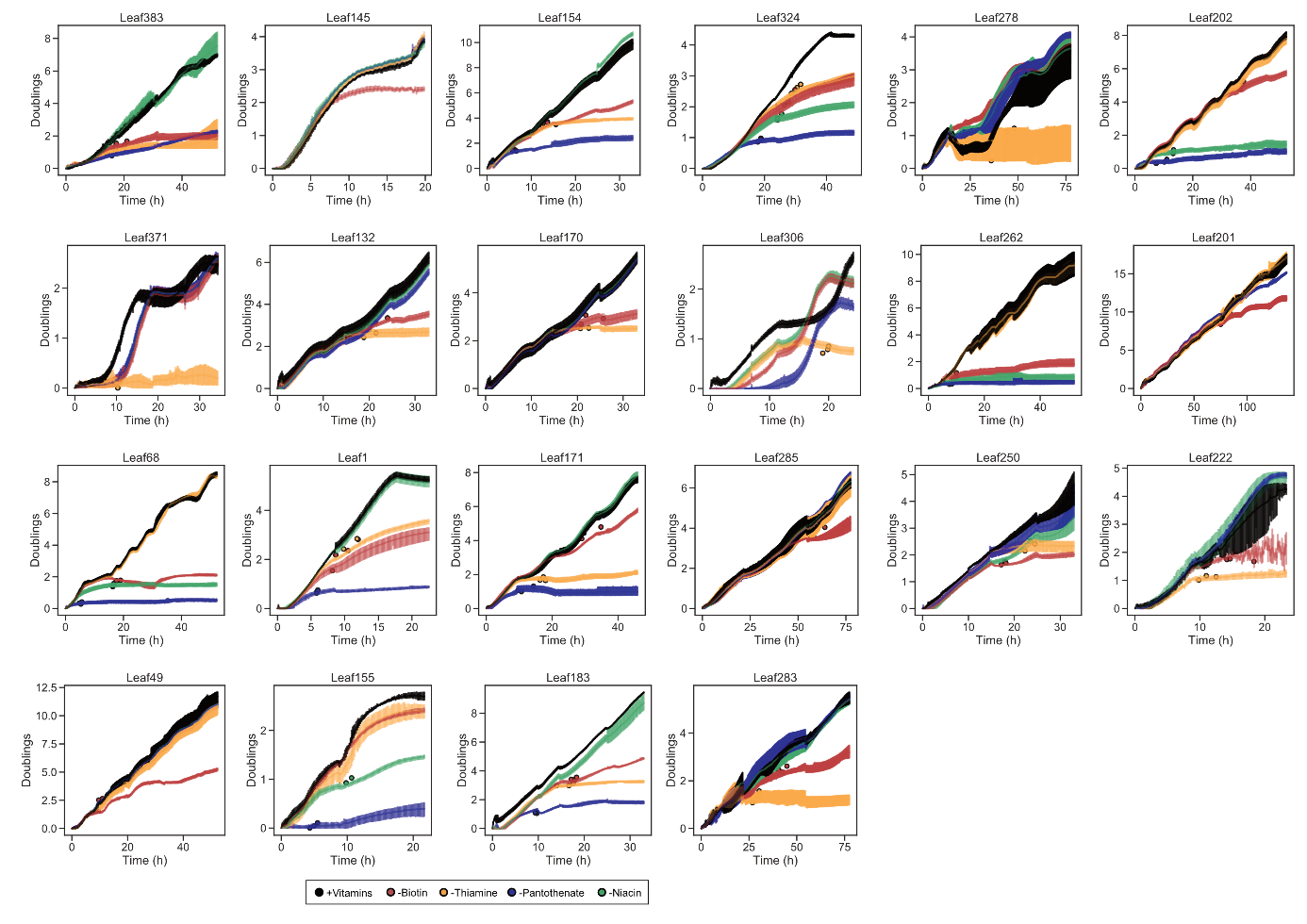


**Supplementary Fig. 2** **Dynamic vitamin depletion experiment.** The storage for each vitamin in each tested strain is indicated in the scatterplot overlay; the scatter is only shown if depletion occurred, and strain was found auxotrophic. Cells were switched to medium free of each vitamin at *t* = 0, and the time point at which vitamin depleted and supplemented cultures deviated are visualized over the lineplot for all three replicates separately. Source data in **Source Data 2**.


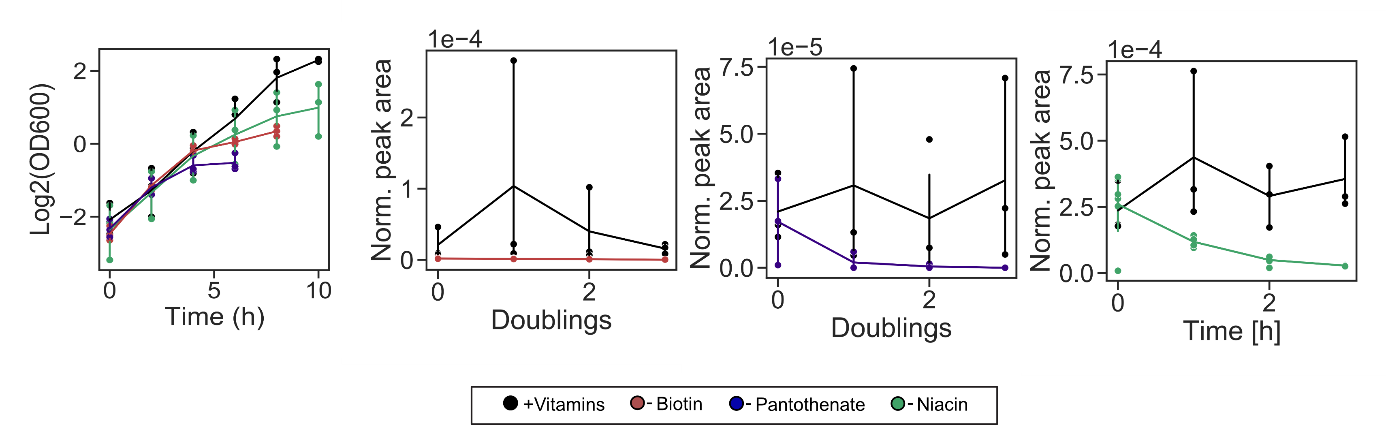


**Supplementary Fig. 3** Depletion of intracellular coenzymes. Cells were switched to medium free of each precursor vitamin at t = 0 and intracellular coenzymes were measured at indicated time points (1/doubling) on a LC/MS metabolomics platform. Model strain was *Rhizobium* Leaf68. Source data in Supplementary Source Data 2.


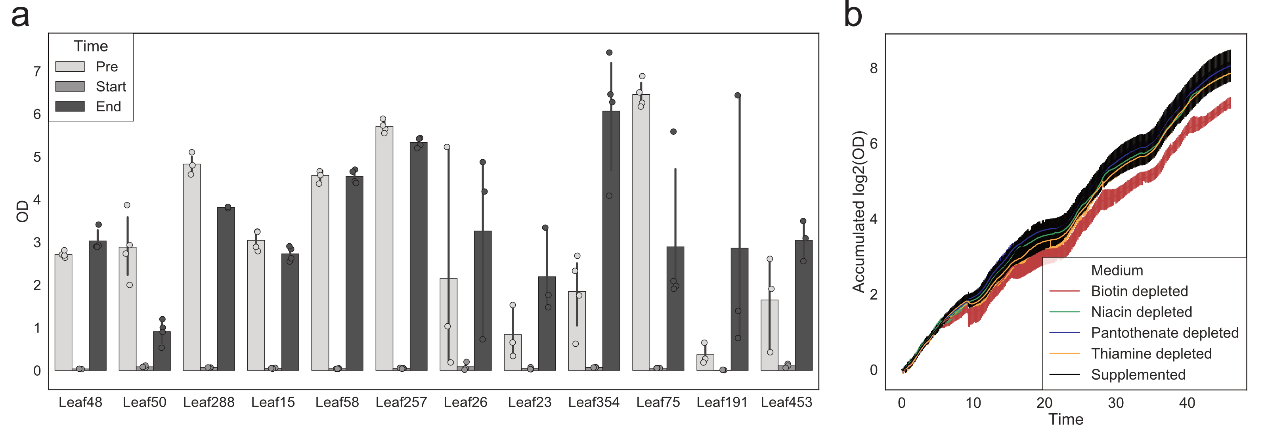


**Supplementary Fig. 4** **Validation of prototrophic strains a)** 12 strains were grown to stationary phase (n = 4 or 3) in supplement-free media (“Pre”). The cultures were diluted and allowed to grow for another 24 hours (“Start” and “End” bars), providing sufficient evidence of ability to grow without supplements (> 10 doublings). Note that for *Serratia* Leaf50, the main culture was terminated after three doublings. Source data in **Supplementary Source Data Fig. 3. b)** *Firmicutes* Leaf13 was found prototrophic due to its ability to maintain exponential growth in a depletion experiment (shown for auxotrophs in **Fig. 1**). Source data in **Supplementary Source Data 4.**


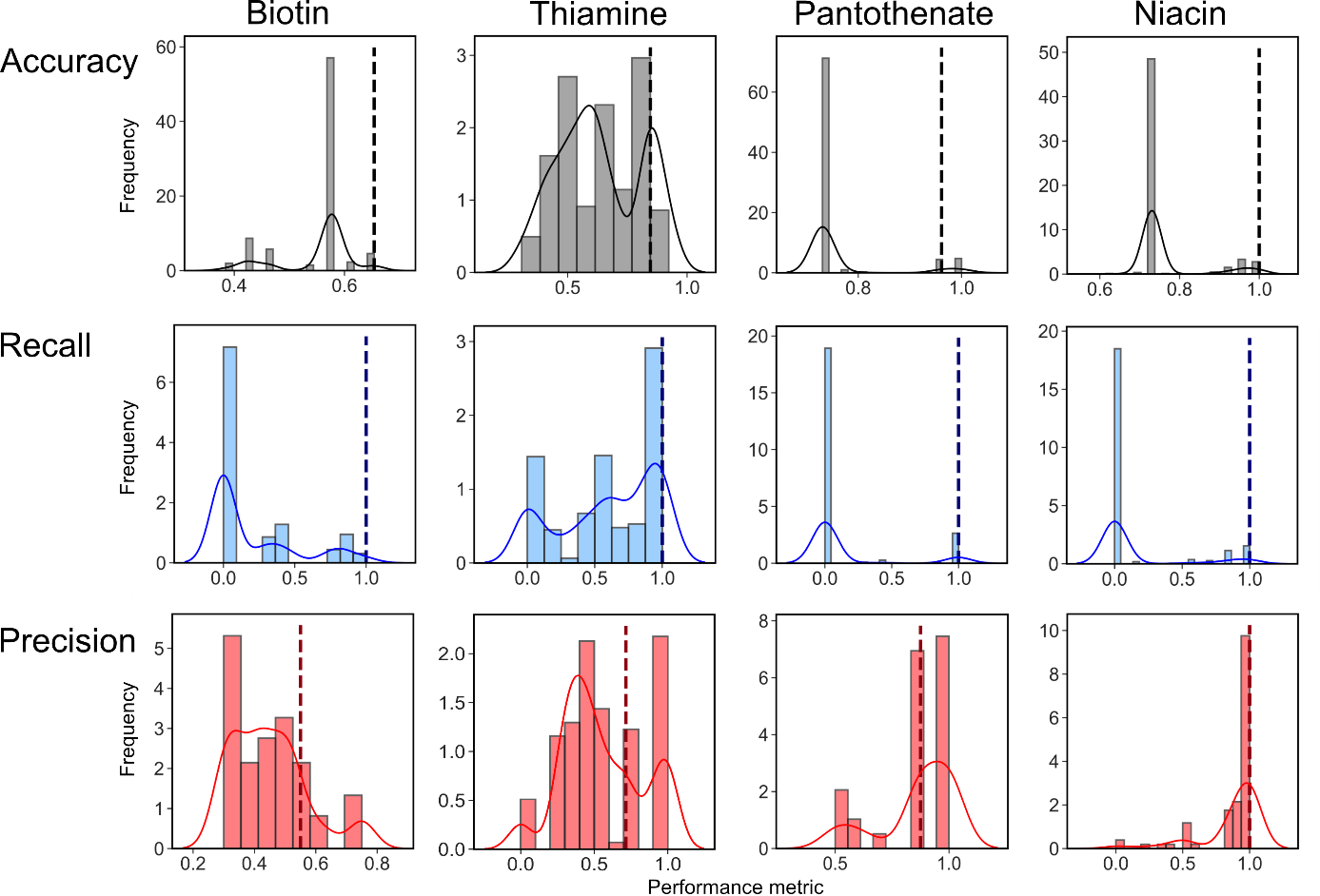


**Supplementary Fig. 5** **Performance of decision tree classifiers in predicting auxotrophy.** In each column, auxotrophs and prototrophs (n = 63, including 35 which were validated and 28 from the screen in **Supplementary Fig. 1c**) for one of the four vitamins were classified using a classifier trained on random features (histogram) or selected (dashed lines) features.


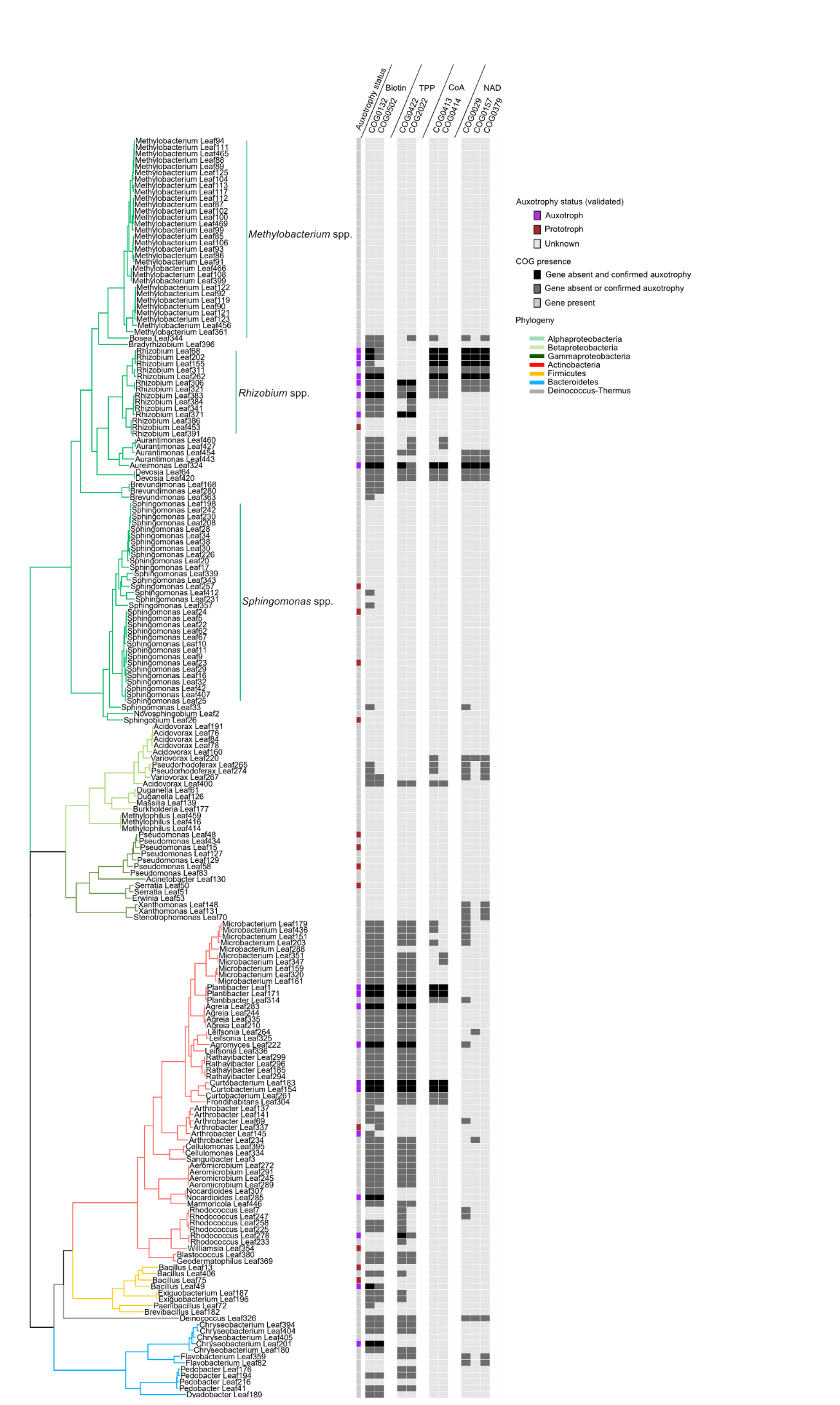


**Supplementary Fig. 6 Phylogenetic analysis of gene absence observed to co-occur with auxotrophy in the At-LSPHERE culture collection.** The tree is colored based on phylum and Proteobacteria are further colored by class; some of the major clades are annotated for clarity. First heatmap column represents auxotrophy status validated in this study (**Fig. 1**, **Supplementary Fig. 4**). For each biosynthetic pathway (Biotin, TPP, CoA, and NAD(P)), each identified gene absence is depicted in a heatmap column representing whether the corresponding COG term was found present or absent in the genetic analysis. Black cells in the heatmaps refer to confirmed auxotroph (respectively for each coenzyme) where the gene was also absent; the gray cells depict strains where either auxotrophy or lack of the depicted gene was observed. The phylogeny was downloaded from phylloR (<https://rdrr.io/github/cmfield/phylloR/man/phylloR.html>)


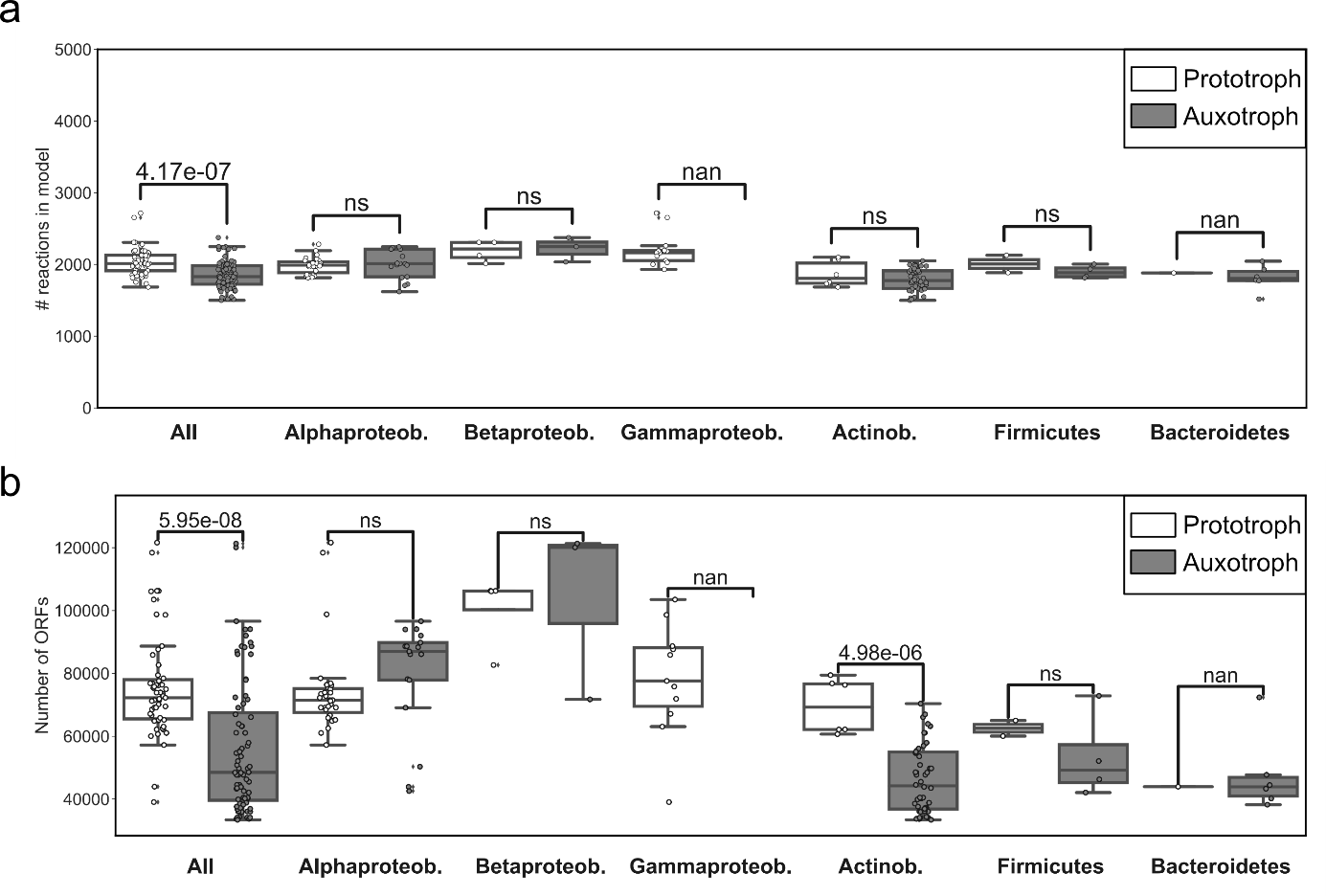


**Supplementary Fig. 7** **Comparison of number of functional genomic units. a)** Genome scale model sizes. Models were automatically generated using Carveme^33^ from RefSeq accession numbers in **Source Data 5.** **b)** Number of predicted open reading frames. In this analysis, any fragment of DNA longer than 120 base pairs and situated between start codon (ATG) and any stop codon (TAA, TAG, TGA) was considered an open reading frame. Genomes were accessed using RefSeq accession numbers in **Source Data 5.**

**
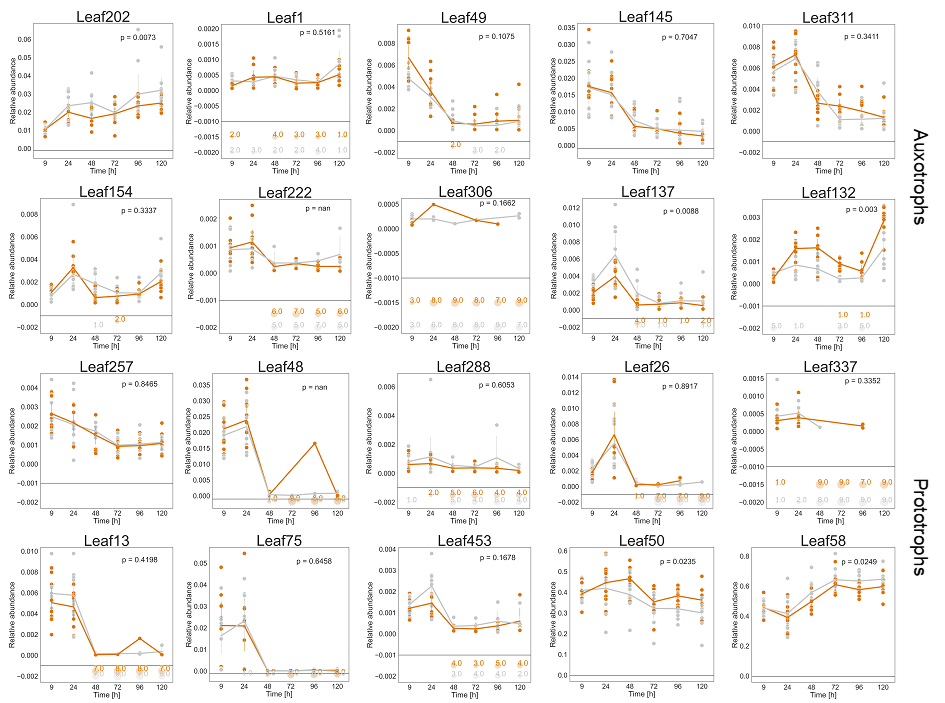
**

**Supplementary Fig. 8 Relative abundance profiles in co-cultures**. Cultures were either supplemented with vitamins (orange) or no vitamins were added (grey). All 20 strains were co-cultured together. The dots below the horizontal line at y = 0.0 indicate the number of cultures in which a given strain was not detected. Source data in **Source Data 6**


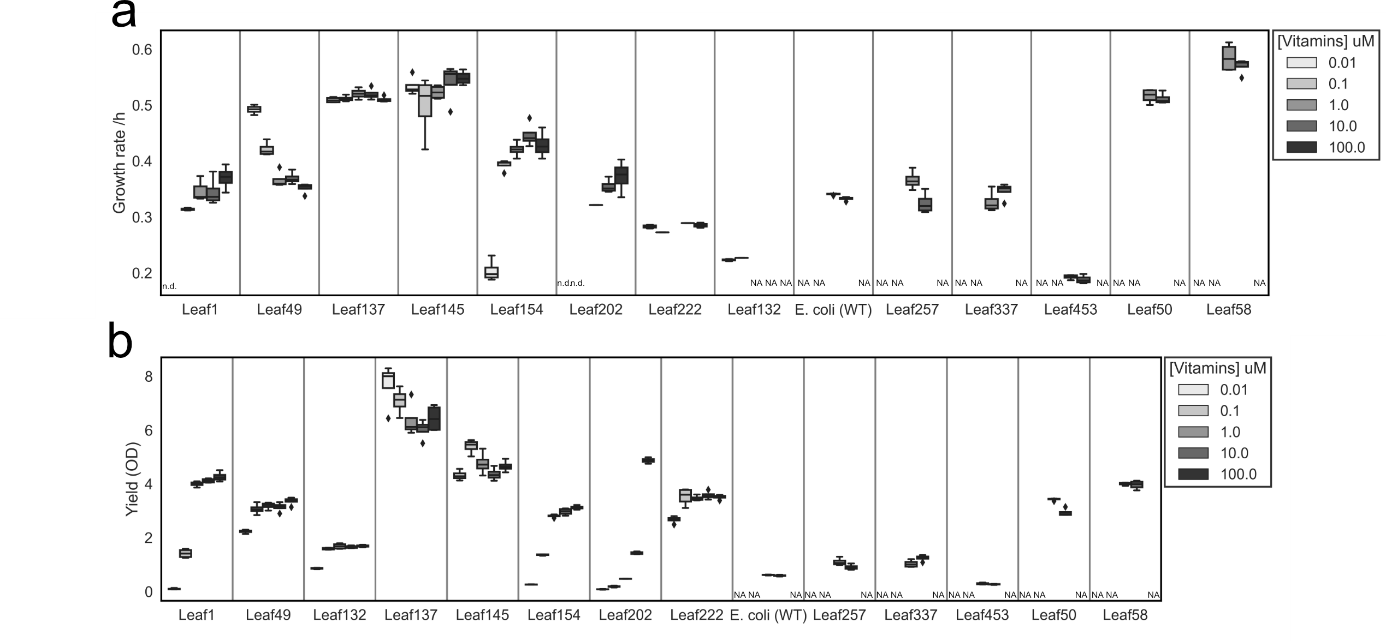


**Supplementary Fig. 9 Growth parameters for selected auxotrophs and prototrophs in increasing vitamin concentration.** Note that 1 µM vitamins were used in all other experiments in this study. n.d. = not detected, NA = not analyzed. **a)** Growth rate. Source data in **Supplementary Source Data 5.**  **b)** Yield. Source data in **Supplementary Source Data 6.**


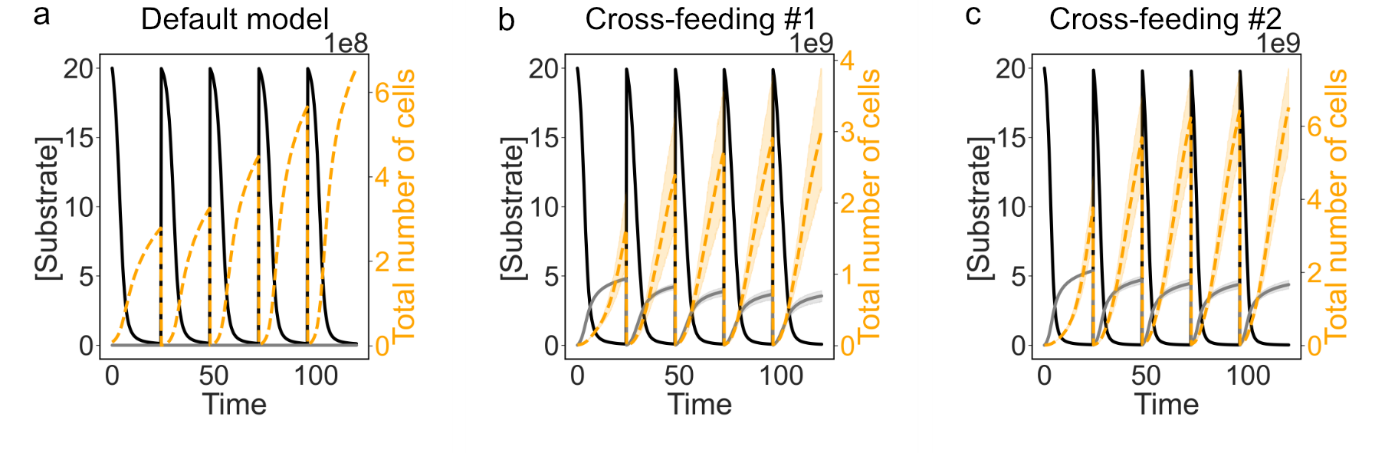


**Supplementary Fig. 10** **Substrate concentration and cell number in Consumer Resource models.** If not stated otherwise, parameters were as follows: Relative abundance threshold for counting as “present”: 5e^-3^, Carrying capacity: yield as parametrized experimentally, Growth rate threshold for by-carbon source secretion: 0.4 h^-1^, substrate affinity for by-product equal between groups (depends on growth rate). Black line corresponds to glucose, the carbon substrate in the medium. Gray line corresponds to the secreted carbon by-products. **a)** Default model where growth parameters were as determined in individual cultures and no cross-feeding terms were added. **b)** Models extended with carbon cross feeding; the carbon secretion thershold was varied between 0.3 h^-1^ (n = 15) and 0.6 h^-1^ (n = 3). **d)** Carbon cross-feeding extension. Efficacy of auxotrophs to preferentially use the second carbon source was varied from 2 to 5 fold that of prototroph


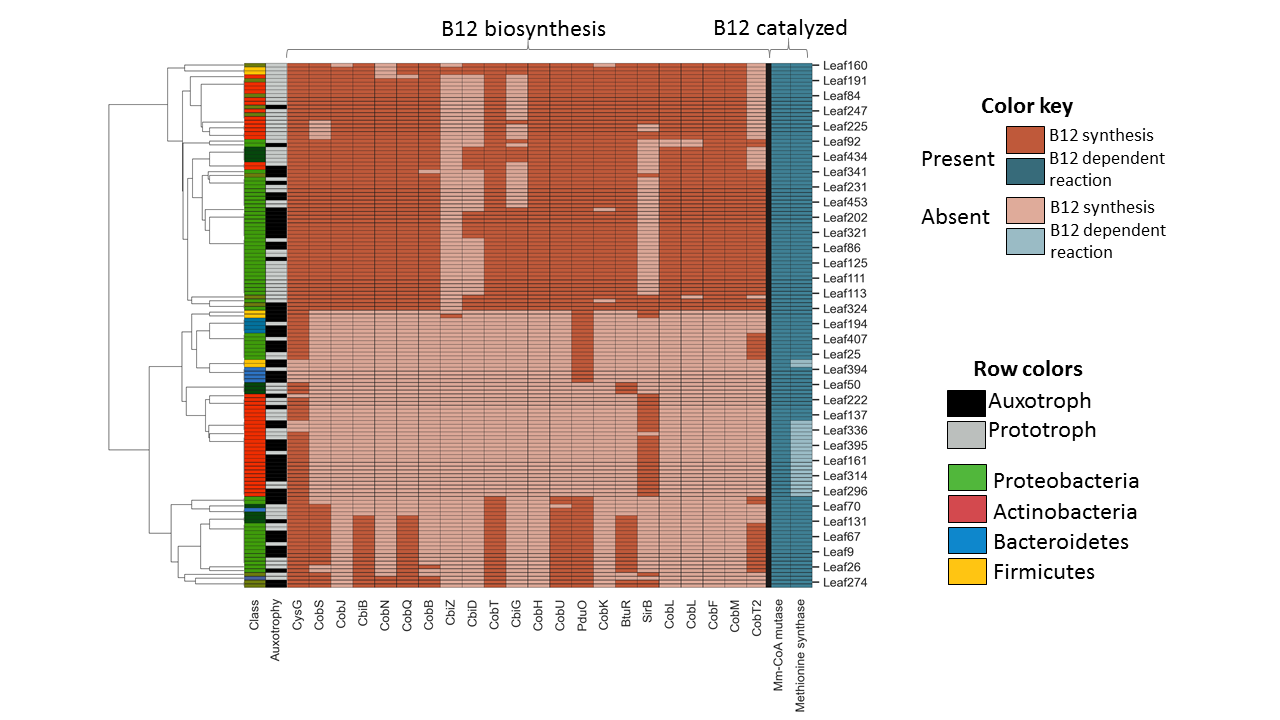


**Supplementary Fig. 11 Presence of vitamin B12 biosynthesis and utilization genes.** Auxotroph/prototroph refers to putative auxotrophy for one of the ten vitamins tested from Supplementary Fig. 1. None of the strains were confirmed vitamin B12 auxotrophic. Compounds made available by the plant represent a way for auxotrophic bacteria to evolve, but the type of auxotrophy would strictly be limited by what the plant can offer. Therefore, apart from asking which auxotrophies occur, we also asked for which vitamins auxotrophies did not occur. Notably, we did not observe any cobalamin (vitamin B12) auxotrophies, despite cobalamin being resource intensive to produce. We verified that the bacteria in the collection encode cobalamin-dependent methylmalonyl-CoA mutases and methionine synthases, and that most of the strains in the community seemingly lacked more than half of the biosynthetic enzymes for cobalamin. Plausible explanations to why *A. thaliana* microbiota does not support cobalamin auxotrophy remain unknown. We speculate that the contributing factors include plants independence of cobalamin, and that it might be less prone to sharing due to its size (both related to crossing the cell membrane and diffusion in the leaf habitat) and its catalytic *modus operandi* as a covalently bound prosthetic group. Source data for COG terms in **Source Data 4** and for auxotrophy predictions in **Supplementary Source Data 1**.

## Supplementary notes

### Supplementary note 1

This supplementary note provides details of the biosynthetic pathways of the four coenzymes for which auxotrophs were identified in this study as well as reaction mechanisms for the biosynthetic enzymes we predict are absent in auxotropic strains. The statistical analysis in which these genes were identified is shown in **Fig. 2** and **Table 1** and in the corresponding results section, and the presence/absence profiles for the At-LSPHERE collection is shown in **Supplementary Fig. 5.**

The first gene loss event we identified pertains to biotin, which is synthesized from a pimelic acid precursor that is cyclized via the addition of CO_2_ to become dethiobiotin by BioD. A sulphur atom is then added to the catalytic site in a step catalyzed by biotin synthase (BioB; COG0502). As all our biotin auxotrophs lacked the gene for dethiobiotin synthetase (*bioD*; COG0132), it can be expected that most auxotrophic strains are able to grow with dethiobiotin supplementation as well as with biotin itself. Thiamine consists of two functional groups: a pyrimidine and a thiazole ring. ThiC catalyzes the formation of the pyrimidine moiety and ThiG the thiazole moiety, respectively – the subsequent steps in thiamine biosynthesis concern the condensation of these two compounds into thiamine. Of the 14 thiamine auxotrophs, 12 were found to lack both *thiC* and *thiG* (COG0422 and COG2022, respectively). One thiamine auxotroph (*Rhizobium* Leaf383) has *thiC*, and one (*Aureiomonas* Leaf324) has *thiG*. Therefore, while almost all (12/14) thiamine auxotrophs are expected to require either both precursors (pyrimidine and thiazole) or fully-formed thiamine, Leaf383 should be able to grow in the presence of thiazole only and Leaf324 should grow in the presence of pyrimidine. Pantothenate, a CoenzymeA precursor, is synthesized via a series of carboxylation steps from either pyruvate or keto-valine. To process pantothenate into fully-formed CoA, pantothenate is appended first with a cysteine and then an adenosine moiety. On pantothenate metabolism, all but one auxotroph lacked the genes *panC* (COG0414) and *panB* (COG0413) for pantothenate synthase and ketopantoate hydroxymethyltransferase, respectively. The only exception was *Rhizobium* Leaf383, which harbors both of these genes; however, whether these genes result in functional enzymes remains unclear, and for this one strain we could not explain the genetic origin of the pantothenate auxotrophy. Lastly, the NAD precursor niacin is synthesized via a pathway where aspartate is first oxidized into iminoaspartate. A pyrimidine ring is then created by fusion of a dihydroxyacetone-phosphate with the iminoaspartate yielding quinolinate, followed by phosphorylation. All niacin auxotrophs lacked all three genes for key enzymes in its biosynthesis: aspartate oxidase (NadB; COG0029), quinolinate synthase (NadA; COG0379), and niacin nucleotide pyrophosphorylase (NadC; COG0157).
